# The impact of rare diseases on the quality of life in paediatric patients: current status

**DOI:** 10.3389/fpubh.2025.1531583

**Published:** 2025-03-24

**Authors:** John Sieh Dumbuya, Cizheng Zeng, Lin Deng, Yuanglong Li, Xiuling Chen, Bashir Ahmad, Jun Lu

**Affiliations:** ^1^Department of Paediatrics, Affiliated Hospital of Guangdong Medical University, Zhanjiang, China; ^2^Department of Paediatrics, The 958 Hospital of the People’s Liberation Army, Chongqing, China; ^3^Hainan Women and Children’s Medical Center, Haikou, China; ^4^Department of Paediatrics, Haikou Affiliated Hospital of Central South University, Xiangya School of Medicine, Haikou, China

**Keywords:** rare diseases, prevalence, disease burden, paediatrics, quality of life

## Abstract

Rare diseases, also known as orphan diseases, are a group of disorders that affect a small percentage of the population. Despite individually affecting a small number of people, collectively, they impact millions worldwide. This is particularly significant in paediatric patients, highlighting the global scale of the issue. This review delves into the exact prevalence of rare diseases among children and adolescents and their diverse impact on the quality of life of patients and their families. The review sheds light on the complex interplay of genetic and environmental factors contributing to these conditions and the diagnostic challenges and delays often encountered in identifying and categorising these diseases. It is noted that although there have been significant strides in the field of genomic medicine and the development of orphan drugs, effective treatments remain limited. This necessitates a comprehensive, multidisciplinary approach to management involving various specialities working closely together to provide holistic care. Furthermore, the review addresses the psychosocial and economic burdens faced by families with paediatric patients suffering from rare diseases, highlighting the urgent need for enhanced support mechanisms. Recent technological and therapeutic advancements, including genomic sequencing and personalized medicine, offer promising avenues for improving patient outcomes. Additionally, the review underscores the role of policy and advocacy in advancing research, ensuring healthcare access, and supporting affected families. It emphasises the importance of increased awareness, education, and collaboration among healthcare providers, researchers, policymakers, and patient advocacy groups. It stresses the pivotal role each group plays in improving the diagnosis, treatment, and overall quality of life for paediatric patients with rare diseases.

## 1 Introduction

Rare diseases (RDs) are considered chronic, progressive, and life-threatening conditions that impact a small percentage of the population, resulting in elevated levels of pain and suffering. These diseases lack effective treatments, and the symptoms exhibit considerable variability among individuals (1, 2). The absence of a universal definition poses a critical constraint in determining the prevalence of rare diseases (3). The lack of a universally accepted definition for rare diseases is the result of both practical challenges and regional economic factors. For instance, the United States applies an absolute number criterion based on the potential profitability of developing orphan drugs, whereas the European Union utilises a relative number criterion that considers its diverse population sizes (4). However, this criterion may not be applicable in areas with varying population sizes or where there are only a limited number of diagnosed rare disease patients. In Latin America, for example, there is no consensus on what constitutes a rare disease (5). Consequently, these differing approaches complicate global efforts to standardize the definition of rare diseases.

Following the adoption of the UN resolution on rare diseases, Rare Diseases International (RDI), a global network representing individuals affected by rare diseases, has played an instrumental role in advocating for a standardized definition of these conditions. Thus, in 2015, RDI proposed a universal definition of rare diseases, aiming to create a more cohesive approach to addressing these conditions across borders. However, despite RDI’s recommendations and the implementation of various global frameworks, each country continues to establish its own criteria for defining rare diseases, primarily based on factors such as disease prevalence, the impact on the population, and the capacity of healthcare systems to provide adequate treatment (6). This lack of uniform adoption has led to challenges in ensuring equitable access to healthcare and treatments for rare disease patients worldwide.

The absence of epidemiological data compounds this challenge, impeding a comprehensive understanding of their incidence and impact on individuals and society. Children with rare diseases face a more complex and uncertain future than children with more common diseases. An estimated 10,000 distinct rare diseases have been identified, with over half afflicting children and contributing to approximately 75% of all cases, primarily driven by genetic mutations accounting for about 80% of cases (7).

Globally, approximately 400 million individuals are impacted by rare diseases, and half of these are children (8, 9). The burden of these diseases encompasses significant implications for patients, their families, and the healthcare system. Furthermore, the absence of efficacious treatments exacerbates the burden, leading to heightened morbidity and mortality. A comprehensive understanding of rare disease burden is imperative for developing effective public health policies and targeted interventions.

## 2 Prevalence

The assessment of rare disease prevalence poses challenges due to significant variability across geographic regions. Recent data indicates that approximately 400–475 million individuals are affected by rare diseases, with an estimated 370–500 million being Indigenous people who encounter difficulties in accessing rare disease diagnosis, treatment, and healthcare services (10). A recent study shows an increase in rare disease prevalence from 10.57 to 33.21 cases per 100,000 individuals, reflecting an average annual rise of 19.46% (11). Nearly 75% of rare disease cases manifest in children, and regrettably, 30% of affected children do not survive beyond the age of five. Furthermore, 80% of rare diseases have been attributed to genetic factors (12, 13). To underscore the impact of these statistics, it is notable that half of all rare disease patients are children, three out of ten afflicted children do not reach their fifth birthday, and genetic mutations account for eight out of ten rare diseases (8, 9). The rise in the prevalence of rare diseases is not because the situation is deteriorating but due to advancements in diagnostic techniques, improved access to healthcare, and heightened awareness of these conditions. For instance, in Taiwan, advancements in diagnostic capabilities have led to an increased identification of rare diseases that were previously underdiagnosed (11). This rising prevalence should not be interpreted as a sign of worsening public health; instead, it represents a positive outcome resulting from enhancements in healthcare systems.

Regionally, a significant portion of the EU population, ranging from 27 to 36 million individuals, is impacted by approximately 8,000 rare diseases, with 50–75% of these cases affecting children (14, 15). In the United States, rare diseases are estimated to affect around 25–30 million people, encompassing one in ten Americans (8, 16). Australia is home to an estimated two million individuals affected by rare diseases, including 400,000 children (17, 18). The exact prevalence of rare diseases in China is undetermined, with some affecting fewer than 140,000 individuals and others impacting up to 49–82 million people (19, 20). Estimates suggest that around 50 million Africans, 40–50 million individuals in Latin America, and 25 million people in the Middle East are affected by rare diseases (21).

A recent systematic review has emphasized the varying prevalence of rare diseases based on disease type - metabolic disorders being the most common subgroup, affecting an estimated 1 in 1,458 individuals, while immunological and haematological disorders are reported as the rarest, with an impact of 1 in 6,000 individuals (22, 23). The lack of comprehensive data on rare diseases presents challenges in understanding their precise prevalence and societal impact, especially in paediatric patients.

## 3 Genetic and environmental factors

Most rare diseases are genetic, resulting from mutations in one or more genes. These genetic mutations can be inherited from parents or occur *de novo*. For example, cystic fibrosis is caused by mutations in the CFTR gene, which can be exacerbated by environmental factors like lung infections, which can worsen the disease (24). In contrast, Duchenne muscular dystrophy is caused by mutations in the DMD gene (25). Some rare diseases result from chromosomal abnormalities, such as deletions, duplications, or translocations. An example is Williams syndrome, caused by a deletion of genetic material on chromosome 7 (26). Many rare diseases are inherited in an autosomal recessive, autosomal dominant, or X-linked manner. For instance, Tay-Sachs disease is inherited in an autosomal recessive pattern (27). Rett syndrome is typically caused by *de novo* mutations in the MECP2 gene on the X chromosome (28). Some rare diseases result from new (*de novo*) mutations that occur spontaneously during the formation of reproductive cells or in early embryonic development. Progeria (Hutchinson-Gilford progeria syndrome) often results from a de novo mutation in the LMNA gene (29). Environmental factors can also play a role in the expression and progression of these diseases, although this is less common. Exposure to certain environmental factors during pregnancy can increase the risk of rare diseases. For instance, maternal infections, substance abuse, or exposure to toxins can result in congenital anomalies (30, 31). Exposure to environmental toxins, such as heavy metals or pesticides, can increase the risk of developing rare diseases. This exposure can occur prenatally or during early childhood. Environmental factors can cause epigenetic changes that influence gene expression without altering the DNA sequence (32, 33). These changes can contribute to the development of rare diseases or affect their progression. In some cases, a combination of genetic predisposition and environmental triggers can lead to the onset of a rare disease. Many rare diseases result from a complex interplay between genetic predispositions and environmental influences. For example, in individuals with a genetic susceptibility to a disease, environmental factors such as diet, lifestyle, and exposures can influence the severity and onset of the condition (34, 35).

Understanding both genetic and environmental factors is crucial for diagnosing, managing, and potentially preventing rare diseases in children. This comprehensive approach helps develop targeted therapies and improve the quality of life for affected individuals.

## 4 Diagnosis

Patients with rare diseases encounter substantial challenges stemming from diagnostic delays. These delays are frequently attributed to initial misdiagnosis, insufficient awareness among healthcare professionals, and restricted access to specialized diagnostic facilities. Prolonged diagnostic timelines can exacerbate the severity of consequences, such as disease progression, irreversible organ damage, and diminished treatment efficacy, underscoring the imperative for heightened medical professional awareness (36, 37). Recent studies have revealed that the average diagnostic journey for rare disease patients exceeds five years (38, 39). Furthermore, another study reported a median duration of 75 months (40). Diagnostic delays and misdiagnosis can be attributed to various factors, including the rare nature of these diseases, which pose challenges in data aggregation, in addition to data heterogeneity and fragmentation with inadequate interoperability, compounded by the absence of a comprehensive repository for rare diseases (41).

A previous study identified inaccessibility and lack of referral as the primary reasons for diagnostic delays, followed by service unavailability and protracted waiting times for affected patients (42). Consequently, the medical journey for rare disease patients entails multiple referrals and extensive testing, occasionally yielding inconclusive diagnoses (37, 43). While common diseases are typically prioritized in the diagnostic process, the low prevalence of rare diseases often results in their being overlooked. Consequently, patients affected by rare diseases must navigate the complexities of their conditions amidst a lack of specialized expertise and knowledge. Furthermore, the absence of targeted health policies contributes to diagnostic delays and restricted access to healthcare services. This combination can lead to significant physical, psychological, and cognitive impairments, and, in certain instances, may result in inadequate or even harmful treatments (36).

The utilization of genomic sequencing has seen a significant rise in the diagnosis of a wide array of diseases, including rare ones (40, 44). In recent years, there has been a paradigm shift in diagnosing rare diseases of epigenetic origin (RDEOs) owing to the transformative impact of next-generation sequencing techniques. High-throughput sequencing has proven effective in detecting and uncovering genetic variants, particularly in rare genetic diseases (8, 32, 45, 46). It has been observed that whole genome sequencing yields a higher diagnosis rate than exome sequencing alone (18, 47, 48). Notable technological progress has significantly enhanced the diagnostic paradigm (Table 1), facilitated by advanced diagnostic tools (49–52). Early screening of newborns is pivotal for the appropriate implementation of medical interventions and future decision-making (53). The combination of single-cell genome amplification technology and deep sequencing holds promise in elucidating the pathogenesis of additional genetic diseases, thereby enabling high-throughput screening and diagnosis of congenital disabilities and rare diseases (54). Long-read RNA sequencing is also applicable in disorders related to rare diseases (8). However, it is imperative to acknowledge the existence of ethical issues and parental concerns surrounding genomic sequencing (55). One of the primary concerns revolves around informed consent. Parents must comprehensively understand the implications of genetic testing, which may involve discovering incidental findings unrelated to their child’s condition. Such findings could include genetic predispositions to other diseases, which can lead to emotional distress and challenging decisions regarding additional testing and disclosure (56, 57). Furthermore, the potential misuse of genetic information—particularly in employment or insurance contexts—raises significant issues of privacy and discrimination that must be thoughtfully considered (58, 59). Parental views on genomic sequencing are diverse. While some parents regard genetic testing as an essential tool for gaining clarity and accessing specific treatments, others express concerns about the psychological burden it may create. The uncertainty associated with genomic sequencing results—whether they will yield a definitive diagnosis or uncover unknown risks—can provoke feelings of anxiety and helplessness (60, 61). In some instances, parents grapple with the moral dilemma of seeking such testing, fearing they may uncover information they are unprepared to handle, mainly when effective treatments are unavailable (58, 62). Noteworthy attention has been directed toward the application of artificial intelligence (AI) in the diagnosis of rare diseases in recent years (63, 64).

**Table 1:** Recent technological and therapeutic advancement in rare diseases.

Technique	Description	Example	Ref
Genetic and genomic technologies			
NGS	NGS technologies enable the sequencing of entire genomes or targeted regions with high throughput and precision	NGS has facilitated the discovery of numerous genetic variants associated with RDs, leading to a better understanding and classification of these conditions; it has also accelerated the identification of novel mutations and rare genetic disorders previously undiagnosed.	Ren [32] and Maver [44]
CRISPR-Cas9 gene editing	CRISPR-Cas9 is a versatile tool for precise gene editing, allowing scientists to target and modify specific DNA sequences base editing involves making targeted changes to the DNA sequence without cutting or disrupting the surrounding genetic material	Preclinical and early clinical trials have shown promising results in treating genetic disorders like sickle cell disease, beta-thalassemia and Gaucher disease the ability to correct genetic defects at their source offers a potential one-time cure for many rare genetic diseases	Pavan [80] and Frangoul [78]
WES and WGS	WES focuses on sequencing the genome’s protein-coding regions WGS sequences the entire genome, including non-coding regions	These technologies have been instrumental in identifying pathogenic variants in patients with complex and undiagnosed conditions, improving diagnostic rates and guiding personalised treatment approaches	Martinussen [18] and Wright [47]
Therapeutic advancements			
Gene therapy	Gene therapy involves delivering functional copies of genes to patients’ cells using viral vectors like AAV	Successful gene therapies, such as Zolgensma for SMA, have demonstrated significant clinical benefits, including improved vision and motor function; ongoing research aims to expand gene therapy applications to other rare genetic disorders	Papaioannou [81] and Jensen [82]
RNA-based therapies	RNA-based therapies include ASOs, siRNA and mRNA therapies	Spinraza (nusinersen), an ASO for SMA, has shown remarkable efficacy in improving motor function and survival; other RNA therapies, like Patisiran (Onpattro) for hereditary ATTR amyloidosis, using siRNA to reduce the production of pathogenic proteins, offering new treatment options for previously untreatable conditions	Mercuri [84] and Zogg [83]
ERT	ERT involves replacing deficient or absent enzymes in patients with lysosomal storage disorders	Treatments like Cerezyme for Gaucher disease and Aldurazyme for MPS I have significantly improved patient outcomes by reducing disease symptoms and slowing progression; new ERTs are being developed for other lysosomal storage disorders, expanding the therapeutic arsenal	Sims [92], Jameson [91], and Ditters [93]
Immunotherapy	Immunotherapy involves using the body’s immune system to fight diseases. This approach has shown promising results in treating various cancers and rare genetic disorders	CAR-T cell therapy uses genetically modified T cells to target and kill cancer cells, such as tisagenlecleucel (Kymriah) for the treatment of ALL	Motte [88]
Advanced drug development			
Orphan drugs	Orphan drug designation incentivises developing treatments for RDs, including market exclusivity, tax credits, and expedited regulatory review	The increase in orphan drug approvals has led to the availability of new treatments for RDs, such as Trikafta for CF; Vyondys 53 for DMD, improving the quality of life and survival rates for many patients	Jones [109] and Servais [110]
Personalised medicine	Personalised medicine tailors treatments based on individual genetics, biomarkers, and phenotypic information	Targeted therapies, such as precision oncology drugs for specific genetic mutations (e.g.; larotrectinib for TRK fusion cancers), offer more effective and less toxic treatment options; personalised medicine approaches are also being explored in rare metabolic and neurological disorders	Drilon [95]
Diagnostic tools			
Advanced imaging technologies	High-resolution imaging techniques, such as 7-Tesla MRI and advanced PET-CT, provide detailed visualization of anatomical and functional changes in rare disease patients	These technologies improve the accuracy of diagnosis, monitor disease progression, and evaluate treatment efficacy in conditions like neurofibromatosis, muscular dystrophies, and rare brain malformations	Hou [49]
Biomarkers	Identification of specific biomarkers for early diagnosis, disease monitoring, and treatment response assessment	Biomarkers like plasma chitotriosidase in Gaucher disease and serum neurofilament light chain in SMA provide valuable insights into disease activity and therapeutic effects, enabling more precise and timely interventions; Genetic testing can identify patients who are most likely to respond to certain therapies	Pawlinski [50] and Kolb [51]
Digital health and telemedicine			
Digital health platforms	Development of mobile apps and online platforms for disease management, symptom tracking, and patient education	Digital health tools empower patients and caregivers by providing real-time data on disease status, improving adherence to treatment protocols, and facilitating remote monitoring by healthcare providers; for example, wearable devices can track vital signs, sleep patterns, and physical activity levels	Gerk [99]
Telemedicine	Expansion of telehealth services, including virtual consultations and remote diagnostics	Telemedicine increases access to specialized care for patients with rare diseases, particularly those in remote or underserved areas; It also supports continuity of care during situations like the COVID-19 pandemic, reducing the need for in-person visits and potential exposure to infections	Senyel [100]
Research and collaboration			
Patient registries and databases	Establishment of comprehensive, multinational patient registries and databases for rare diseases	These resources facilitate data sharing, enhance understanding of disease epidemiology, and support the development of new treatments by providing valuable insights into natural history, clinical outcomes, and treatment responses	Lavelle [68]
Collaborative research initiatives	Collaborative efforts accelerate the discovery of new therapies and diagnostics by pooling expertise, resources, and data	Global research consortium and collaborations, such as the IRDiRC and ERNs; these initiatives also promote harmonized regulatory frameworks and improve patient access to clinical trials and innovative treatments; EHRs are being used to improve data sharing and coordination between healthcare providers	
Emerging therapies and innovations			
Gene silencing techniques	Techniques like RNA interference (RNAi) and ASOs can silence disease-causing genes	These therapies, such as Onpattro (patisiran) for hereditary ATTR amyloidosis Spinraza (nusinersen) for SMA, provide new avenues for treating previously intractable genetic disorders by targeting the underlying molecular mechanisms	Adams [85] and De Vivo [94]
CAR T-cell therapy	CAR T-cell therapy involves engineering a patient’s T cells to recognize and attack specific cancer cells	CAR T-cell therapy’s success has spurred research into its application for rare cancers and other conditions, potentially transforming treatment paradigms; Other CAR-T cell therapies are being developed for various types of RDs, such as MG	Aghajanian [86]
Stem cell and regenerative therapies	Use of stem cells to repair or replace damaged tissues and organs	Stem cell therapies are being investigated for various rare diseases, including HSCT for genetic immunodeficiencies and MSC therapy for rare neurodegenerative disorders	Hoang [87]
Targeted molecular therapies	Development of small molecule drugs that specifically target disease-causing proteins or pathways	Targeted therapies, such as kinase inhibitors for specific genetic mutations in cancer and enzyme inhibitors for metabolic disorders, offer precision treatment options with fewer side effects than traditional therapies	Staedtke [89] and De Boeck [90]

NGS, next-generation sequencing; CAR T-cell, chimeric antigen receptor T-cell; ERT, enzyme replacement therapy; MPSI, mucopolysaccharidosis I; ALL, acute lymphoblastic leukaemia; IRDiRC, international rare diseases research consortium; ERNs, European reference networks; EHRs, electronic health records; HSCT, haematopoietic stem cell transplantation; MSC, mesenchymal stem cell; WES, whole-exome sequencing; WGS: Whole genome sequencing; AAV, adeno-associated virus; ASOs, antisense oligonucleotides; siRNA, small interfering RNA; MRI, magnetic resonance imaging.

Establishing a Clinical Decision Support System (CDSS) utilising Information Retrieval (IR) to aid in the diagnosis of Rare Diseases (RD) presents specific challenges (65). These challenges include catering to physicians’ precise informational needs and ensuring the involvement of domain experts in user-centric development and evaluation (66). Subsequent research endeavors are essential to assess the practical utility of these CDSS in clinical settings. Although tools and resources exist to decipher rare disease variants, aiming to expedite diagnosis and the implementation of timely management strategies, challenges arise in the comprehensive and accurate adoption of specific diagnostic methodologies and in appraising their cost-effectiveness (67, 68). Furthermore, parents encounter difficulties in comprehending the significance of these diagnostic parameters, and reservations and uncertainties exist concerning the application of select diagnostic tools in paediatric cases (69–71).

## 5 Treatment

There is currently no effective treatment for most rare diseases. Out of the 10,000 reported rare diseases globally, only 5% can be managed with pharmaceutical interventions, underscoring the critical need to enhance patient access to orphan drugs (72). Due to the scarcity of these conditions, the requisite expertise for their management is often limited, resulting in numerous undiagnosed cases and suboptimal treatment regimens among children with rare diseases (73). The rarity of these diseases also complicates the recruitment of an adequate number of patients for clinical trials, posing challenges to the development of effective treatments and cures for many rare diseases (74, 75). Additionally, issues such as low prevalence, limited knowledge of disease progression, and phenotype heterogeneity hinder drug development for rare diseases (76). Other contributing factors include disease severity and the absence of therapeutic alternatives (77). Meanwhile, inadequate healthcare services, unstructured or complex healthcare facilities, and bureaucratic policies hinder access to appropriate medical care or orphan drugs in certain regions. Nonetheless, advancements in prenatal diagnosis and screening hold promise for enhancing diagnostic accuracy and formulating preventive and treatment strategies for paediatric patients with rare diseases (54).

Significant progress has been made in the treatment of rare diseases, particularly those resulting from genetic mutations (Table 1). Advancements encompass CRISPR/Cas9-based gene therapy (78–80), gene therapies (81, 82), RNA-based therapies (83, 84), genetic silencing techniques (85, 86), stem cell therapies (87), immunotherapy (88), molecular-targeted therapies (89, 90), and enzyme replacement therapies (91–94). Personalized medicine is pivotal in addressing rare diseases, with multidisciplinary collaboration holding the potential for tailoring treatments to specific rare diseases (95–97). For instance, there is notable progress in the personalized treatment of Batten’s disease using nusinnersen (98). Additionally, digital health platforms and telemedicine are being explored to expand patients’ treatment accessibility (99, 100).

These developments mirror a rapidly evolving landscape in the rare diseases sphere initiated by state-of-the-art technologies and collaborative endeavors. They offer the prospect of enhancing treatments and overall quality of life for rare disease patients, ushering in renewed hope and improved outcomes. However, their application in paediatric patients with rare diseases remains to be explored.

In addition to traditional funding models for orphan drugs, alternative approaches such as value-based healthcare (VBHC) are increasingly gaining attention (101, 102). Under a value-based model, treatment costs are directly linked to the outcomes achieved, promoting a more efficient allocation of resources and ensuring both efficacy and accessibility of treatments. This model can potentially enhance access to orphan drugs, particularly in resource-constrained settings where the high costs of these treatments can pose significant barriers (103, 104). By focusing on the clinical benefits and long-term outcomes associated with rare disease treatments, value-based healthcare may provide a sustainable solution to the financial burdens linked to rare disease care. Studies, such as those by Fuchs et al., have demonstrated promising results in treating sarcoma patients (105). Furthermore, several countries have reported similar encouraging outcomes when adopting these models (106).

Thus, VBHC signifies a profound transformation in the way healthcare systems approach the management of rare diseases. By prioritising patient-centred outcomes and encouraging collaborative practices, VBHC has the potential to enhance both the quality and effectiveness of care for individuals facing the unique challenges posed by rare conditions. Although challenges in implementation persist, a steadfast commitment to value-based principles can lead to significant improvements in patient outcomes, ultimately benefiting not just individuals but society as a whole (107).

## 6 Orphan drugs

The pharmaceutical industry’s historically limited focus on orphan drugs has shifted, with a growing recognition of their importance due to market dynamics and technological progress (16). This evolving landscape has prompted diverse business strategies in this expanding sector (108). Orphan drugs are indispensable therapeutic options for individuals with rare conditions, such as cystic fibrosis (CF) (109) and Dunne muscular dystrophy (DMD) (110). Nevertheless, their notably higher costs compared to non-orphan drugs have prompted inquiries into their cost-effectiveness and accessibility. For instance, expenditure on orphan drugs in the United States has surpassed 10%, a figure five times greater than that of non-orphan drugs (111). The utilization of respiratory syncytial virus (RSV) prophylactic programmes for CF patient care generated specific incremental cost-effectiveness ratios and annual budget impacts (112). Treatment of spinal muscular atrophy (SMA) patients with particular drugs led to increased annual costs (113). Consequently, the pricing of select rare disease drugs exceeds international reference prices, rendering most drugs financially unattainable for rare disease patients (114, 115). Apart from the elevated cost of certain orphan drugs, challenges persist in conducting clinical trials for orphan drug research (116).

Notwithstanding these high-cost challenges, several case studies highlight the potential for positive outcomes in the treatment of rare diseases through the application of innovative interventions. For example, the development of CFTR modulators, such as Ivacaftor and Orkambi (Lumacaftor/Ivacaftor), has significantly transformed the treatment landscape for children with cystic fibrosis (CF). Research has shown that these medications improve lung function and nutritional status, resulting in a better quality of life and fewer hospitalisations (117). Another important case is Nusinersen (Spinraza), an antisense oligonucleotide approved for the treatment of spinal muscular atrophy (SMA) in children. Clinical trials have provided evidence that Nusinersen can significantly enhance motor function in infants diagnosed with SMA, contributing to greater independence in their daily activities, enabling them to reach developmental milestones that would have been impossible without treatment (118). Additionally, Idursulfase (Elaprase) represents an enzyme replacement therapy developed for Hunter syndrome (Mucopolysaccharidosis Type II), further illustrating the positive impact of innovative treatments for rare diseases. Clinical studies have shown that idursulfase can enhance walking ability, reduce organ enlargement, and improve the overall quality of life for affected children (119). The approval of these therapies marks a significant advancement in the field of orphan drug development, demonstrating the effectiveness of targeted treatments in enhancing patient outcomes.

Patient organizations and advocacy groups have been instrumental in raising awareness and advocating for access to treatments such as Dornase alfa and Ivacaftor, spearheaded by the Cystic Fibrosis Association of South Africa, which have significantly improved diagnosis rates and the management of cystic fibrosis (CF) patients, resulting in better lung function and a higher quality of life (120). Moreover, the Brazilian Ministry of Health developed a national screening programme for phenylketonuria (PKU), which included access to dietary products and healthcare support for affected families. This initiative has successfully reduced the incidence of cognitive disabilities among treated individuals, highlighting the potential of targeted public health strategies to markedly improve health outcomes (121).

## 7 The burden of rare diseases in paediatric patients

Rare diseases can significantly impact the quality of life of individuals, leading to physical, emotional, and social stress that detrimentally affects their daily functioning and overall well-being (122–124). The scarcity of these diseases often results in challenges for patients in accessing specialized medical care, treatments, and support services. Furthermore, many rare diseases carry a heightened risk of morbidity and mortality as a result of inadequate and unavailable treatments (124).

The United Nations (UN) recognises the disproportionate impact of rare diseases on individuals, particularly in relation to poverty, discrimination, and work-related adversities (125). This discrimination can significantly affect young individuals, notably in terms of education and social integration. Efforts have been made to establish an economic framework aimed at ameliorating the burden of rare diseases, along with the regulation of orphan drug usage in children (126–128). Yet, individuals living with rare diseases may still face enduring psychological, social, and economic vulnerabilities, encountering specific challenges in education, employment, and social engagements.

### 7.1 Financial burden

The economic ramifications of rare diseases are substantial, often resulting in additional healthcare expenses and financial hardships for affected families (129–132). Research by Yang et al. (133) in the United States disclosed an estimated total economic burden of $997 billion for 379 rare diseases in 2019. This encompassed direct medical costs of $449 billion (45%), indirect costs of $437 billion (44%), nonmedical costs of $73 billion (7%), and healthcare costs not covered by insurance amounting to $38 billion (4%). In Europe, the estimated expense stood at 147 billion euros, constituting 7.2% of the total pharmaceutical spending (21). The economic burden of rare diseases in Latin America varies across different countries (134). Presently, data are absent regarding the estimated costs of rare diseases in Africa (135). Heinrich et al. (136) put forward an estimated combined cost of €316,990 per patient diagnosed with three distinct rare diseases. Treatment of Fabry disease with enzyme replacement therapy (ERT) escalated the annual economic burden to €9.9 million (137). The estimated economic cost of spinal muscular atrophy (SMA) amounted to CAD$51,796 per patient annually (138), while costs for haemophilia varied across studies (139, 140). López-Bastida et al. (141, 142) reported distinct costs for systemic sclerosis.

The costs associated with rare diseases (RDs) treatment in paediatric patients can present a significant financial burden. The estimated expenditure for paediatric patients is reported at HK$840,908 (US$107,809), which is notably higher than the HK$324,126 (US$41,555) cost for adult patients (123). For instance, the treatment expenses for cystic fibrosis (CF) can escalate up to $300,000 per annum, rendering it one of the most economically demanding chronic diseases within the healthcare spectrum (143). A study conducted in Germany highlighted an approximate mean cost of €17,551, contributing to a national burden of €159 million (144). Additionally, research conducted by Thorat et al. (145) has revealed that annual costs for commercially insured children with CF encompass a wide range: $6,500 to $53,678 for hospitalizations, $20,373 to $47,494 for outpatient prescriptions, and $9,341 to $19,433 for outpatient visits per child. Medicaid-insured children, on the other hand, incur costs ranging from $10,460 to $64,327 for hospitalizations, $13,265 to $45,082 for outpatient prescriptions, and $7,137 to $9,875 for outpatient visits, compared to $5,000 in the comparison group. These findings underscore the substantial financial impact of treating CF patients, even with insurance coverage. Various studies have presented the average annual costs for treating different medical conditions. Specifically, spinal muscular atrophy (SMA) incurred an estimated average total yearly cost of $143,705 per household, with all SMA types incurring an average annual indirect healthcare cost of $63,145 (146). López-Bastida et al. reported an annual average cost of €33,721 for SMA patients (147), while in Germany, the average annual cost of SMA per patient was estimated at €70,566 (148).

The annual economic cost of Duchenne muscular dystrophy (DMD) was estimated at €70,000 in Italy (25) and $46,706 in Australian dollars for children with DMD in Australia (149). For Gaucher disease, the annual economic burden amounted to $48,771, with an individual yearly productivity loss of $1,980 (150) and $3,440,199.2 in India (151). The total annual cost of treating children with beta-thalassemia was estimated at US$127,535 (152), while for transfusion-dependent thalassemia, the cost was estimated at $606,665 (153). Lastly, for Prader-Willi syndrome (PWS), the mean costs were estimated at €58,890 per individual in France (154) and $28,712 in the USA (155).

Thus, the economic impact on paediatric patients with distinct rare diseases varies across countries. Concerns regarding high drug prices and their influence on healthcare expenditure are also prominent (156). While some of these economic costs are not specific to children, given the elevated occurrence of rare diseases in the paediatric population, it is reasonable to surmise that the primary incidence of these costs is among paediatric patients. For illustration, the average hospital cost for treating cystic fibrosis in children amounted to US$26,249.23, in contrast to US$21,600.91 for adults (157). Similarly, Kodra et al. documented a cost of €140,637.30 for children with Cri du Chat syndrome, in comparison to €40,353.25 for adult patients (158), while the medical expenditure for treating phenylketonuria (PKU) was $73,204 in children compared to $40,705 for adult patients (159).

### 7.2 Physical burden

Numerous rare diseases result in significant physiological dysfunction, leading to chronic pain, fatigue, and decreased mobility, which markedly impede a child’s daily activities (160). Moreover, many rare diseases pose a higher risk of morbidity and mortality compared to common diseases due to the inadequacy and scarcity of treatments (124). Despite improving CF treatment, patients still face physical difficulties (161). Previous research has demonstrated that over 70% of individuals with a rare disease encounter difficulties with daily activities, tasks, motor/sensory functions, and social interactions. Furthermore, more than 42% of patients and caregivers dedicate two hours daily to illness-related tasks such as hygiene, treatment management, housework assistance, and patient mobility (162). Individuals with rare diseases and employed caregivers have specific needs that employers do not consistently address. The ability to request special leave represents a significantly unmet requirement for rare disease patients, as 41% sought leave but did not secure it. The most challenging period pertains to seeking a diagnosis, a process that can encompass approximately six years or longer (163).

### 7.3 Emotional burden

Rare diseases and their associated symptoms can significantly impact the psychological well-being of children and their families. The profound social isolation experienced by many children with rare diseases often leads to elevated levels of depression, anxiety, and low self-esteem (14). Furthermore, the reliance on medical devices, such as oxygen tanks, can engender feelings of burden and dependency (160).

Families of children with rare diseases also endure considerable emotional strain while navigating the intricate healthcare system, which frequently involves expensive treatments and procedures not covered by insurance (163, 164). Caregiver burden is a common experience as families balance caregiving responsibilities with work and other obligations, often resulting in financial challenges that hinder their quality of life (165, 166). A recent study examining the impact of caring for patients with rare diseases revealed that most respondents (97%) reported caring for children with undiagnosed diseases, with 57% having treated ten such patients throughout their clinical careers. Additionally, almost all paediatricians surveyed (98%) encountered difficulties in caring for children with rare diseases due to delayed diagnosis and a lack of available treatments and clinical guidelines (167).

Caring for a child with a rare disease necessitates a shift in the parental role, leading to emotional stress for the parent/caregiver and behavioral adjustments, including medication administration, data recording, medical appointment attendance, and meeting participation (168). Collectively, these factors place a strain on patients, their caregivers, and healthcare professionals, contributing to negative emotional burdens.

### 7.4 Social burden

Rare diseases place a financial strain on families and lead to significant social consequences. The stigma associated with having a rare disease can result in social isolation and discrimination, profoundly impacting children’s educational and social development (169, 170). This social ostracism is correlated with a decreased quality of life and increased psychological distress. Many families feel alienated from their communities due to a lack of understanding regarding the complexities of rare diseases (171). A study conducted in Australia surveyed individuals aged 12 to 24 to assess their health and sense of belonging. The results indicated a strong correlation between belonging to marginalized groups and an increase in chronic health issues, heightened psychological distress, and instances of illness or injury that led to absenteeism from school or work (172). This stigma can impede opportunities for social engagement, resulting in emotional distress for both the affected individuals and their families. Children with rare diseases frequently face challenges that affect their academic performance and involvement in school activities, primarily due to the necessity of attending regular medical appointments (173). The emotional impact of living with a rare disease, coupled with concerns about potential discrimination from peers, can result in feelings of isolation and lowered self-esteem among these children. Furthermore, the uncertainty that accompanies rare diseases often leads to emotional distress for both patients and their caregivers. Parents commonly experience anxiety regarding their child’s diagnosis, prognosis, and the availability of effective treatments, which exacerbates their psychological burden. The struggle to manage caregiving duties alongside personal and professional commitments can further strain family dynamics (174–176). Consequently, patients with rare diseases are at a higher risk of social marginalization compared to those without such conditions, resulting in both physiological and psychosocial trauma, thereby increasing the overall social burden on patients and their families.

## 8 Impact on quality of life

The impact of rare diseases on patients’ quality of life (QoL) is pervasive and often profound. Individuals with rare diseases frequently endure considerable morbidity and mortality, leading to a reduced life expectancy (177, 178). In the case of paediatric patients, they may encounter health-related stigma, bullying, and mental health challenges (174). The intricate nature of rare diseases, delayed diagnoses, and the absence of effective treatments often engender feelings of isolation and psychological distress among patients and their families (126, 179). Assessing health-related quality of life in rare diseases presents unique challenges, but innovative approaches can enhance measurement and clinical outcomes (179). Numerous studies have demonstrated that children with rare diseases exhibit lower quality of life scores than children without chronic diseases (161, 170, 180). As delineated earlier, these diminished quality of life scores are frequently associated with the physical, emotional, and social burdens accompanying rare diseases.

Moreover, the impact of rare diseases on families and caregivers should not be underestimated, as caring for a loved one with a rare disease can be emotionally and financially taxing, often necessitating significant investments of time and resources (131). Parents tending to a child with a rare disease often harbor unmet needs, encompassing dissatisfaction with healthcare professionals’ knowledge, lack of support, financial hardships, social seclusion, and emotional strain (181, 182). Many of these children are susceptible to short- and long-term neurodevelopmental sequelae that can compound adverse psychological quality of life outcomes.

### 8.1 Physical QoL

Numerous studies have demonstrated the adverse impact of rare diseases on affected children’s physical quality of life. A study focusing on children with rare and undiagnosed diseases revealed that a majority of these individuals experienced limited mobility, leading to difficulties in performing fundamental tasks such as dressing and grooming (183). Similar findings emerged in studies on children with epidermolysis bullosa (184, 185), DMD (186), and Prader-Willi syndrome (187), all of which exhibited decreased physical functioning. While the participation of children with rare diseases in educational settings is deemed advantageous, it presents challenges for the education system and the families involved (173, 188). Moreover, the professional lives of individuals impacted by rare diseases are also significantly affected, with approximately 70% of patients and caregivers having to curtail their professional activities due to factors such as fatigue, memory impairments, time constraints, and commuting difficulties (162).

### 8.2 Emotional QoL

Children impacted by rare genetic disorders commonly experience anxiety and depression, leading to a diminished quality of life. These children often face increased feelings of loneliness, isolation, and low self-esteem due to their condition (14, 189). Families of affected children undergo emotional turmoil, including feelings of sadness, fear, and frustration. The financial burden associated with treating rare diseases further compounds the challenges faced by affected families (178, 188). Furthermore, studies investigating depression and anxiety in patients with cystic fibrosis (CF) and their caregivers revealed significant increases in depressive symptoms among adolescents, adults, mothers, and fathers. Anxiety levels also rose notably across these same groups (190). Additionally, research has shown a compelling connection between sleep disturbances, depression symptoms in children, and diminished health-related quality of life (HRQOL) in adolescents with CF (180).

Moreover, a study examining the sleep quality of caregivers of patients with Dravet syndrome (DS) found severe sleep impairment in caregivers, often associated with patient anxiety, comorbidities, and sleep disturbances. Notably, caregivers of patients with DS exhibited exacerbated sleep quality issues, anxiety, and depression compared to the general population (191, 192). These findings underscore the significant emotional and psychological toll faced by patients and caregivers of individuals with rare diseases.

### 8.3 Social QoL

Patients with rare diseases often rely on patient organizations to ameliorate their circumstances and enhance their quality of life. Facilitating active participation in decision-making processes among patients and their families is imperative to mitigate social exclusion (30). The social exclusion linked to rare diseases can diminish affected children’s social quality of life. Several studies have indicated that children with rare diseases exhibit significantly lower levels of social functioning compared to their peers without chronic ailments (188, 193). Moreover, individuals may experience feelings of worthlessness, disadvantage, rejection, stigmatization, and a loss of purpose due to their condition (194). This social exclusion may further curtail involvement in educational and recreational activities, thereby limiting opportunities for social interaction and integration (172). Furthermore, patients with rare diseases often encounter challenges in securing school accommodations, experience absenteeism, and face disruptions in their social and personal lives (178, 195). The compounding of social exclusion is exacerbated by insufficient communication and weak coordination among educators responsible for children diagnosed with rare diseases (196, 197).

Stigma and discrimination represent significant emotional and social challenges for children living with rare diseases. The social stigma associated with these conditions, especially those with visible symptoms, can lead to discrimination and exclusion in social, educational, and healthcare contexts (173). Children with rare diseases are particularly vulnerable, facing difficulties in schools such as bullying, inadequate accommodation, and missed educational opportunities, all of which could hinder their long-term social development (198). In educational settings, these children may struggle to receive appropriate accommodation due to teachers’ unfamiliarity with their conditions. This lack of inclusion can lead to diminished self-esteem and feelings of alienation, which can further impact their academic performance and social growth (199). The repercussions of social exclusion and stigma may be even more pronounced in resource-limited environments (200).

The emotional and social effects of rare diseases on children are intricate and significantly shaped by stigma and discrimination. These challenges often result in feelings of isolation and stress, which can impede their educational and social opportunities and adversely impact their psychosocial well-being (197, 201). Gaining insight into the lived experiences of these children underscores the necessity for comprehensive awareness campaigns and training for educators aimed at fostering inclusivity within schools. By confronting the stigma associated with rare diseases, stakeholders can enhance educational practices and support systems that empower children to flourish academically and socially, thereby alleviating the barriers they face daily. Through collaborative efforts, experiences of stigma can be transformed into opportunities for understanding, acceptance, and community cohesion, ultimately paving the way for a brighter future for children affected by rare conditions (199, 202).

### 8.4 Parental/caregiver burden and quality of life

Despite the growing attention toward patients with rare diseases, insufficient focus has been placed on the role of parents as caregivers for affected children within the healthcare system and health research. The impact of rare diseases in childhood and adolescence on the psychosocial dynamics of a family is substantial, as these conditions necessitate a high level of care and disease management from the affected families (193, 203). A recent study showed that 45.5% of caregivers reported feelings of care overload, 43% reported coping poorly due to stress, as well as experiencing anguish toward the role of caregiving for children with RDs. While most respondents declared that their entire life was subordinated to the role of caregiver (62.5%), 60.5% felt others did not understand them, and 51.5% believed that their needs were unimportant to others (204).

Variations exist within families regarding the care provided to children with chronic or rare illnesses, which can significantly influence the quality of life of affected patients, contingent upon parental involvement (193, 205). A systematic review indicates that parents of children with rare diseases experience a compromised quality of life compared to standard values and parents of children with other chronic conditions. Results reveal that 75.6% of participants encountered stigmatization, with nearly half (46.4%) reporting instances of bullying indicative of low to moderate self-care skills in social activities and peer relationships. Additionally, 43.9% highlighted mental health issues in their children, while some parents expressed dissatisfaction with the efforts of educators in addressing bullying (174, 175).

A recent systematic review has elucidated the increased economic burden on caregivers of individuals with cystic fibrosis, as well as the lower quality of life utility scores, elevated prevalence of depression and anxiety among caregivers, and reduced caregiver productivity (206). Similarly, caregivers of boys with Duchenne muscular dystrophy (DMD) encounter comparable economic burdens and diminished quality of life (207). Parents of children with chronic illnesses demonstrate a decreased psychological and physical quality of life compared to parents of healthy children. Moreover, caregivers of patients with DMD appear to exhibit prolonged longevity owing to enhanced treatment and management strategies (129, 186), increasing parents’ or caregivers’ burden, thus impacting their quality of life. Caregivers of patients with Dravet syndrome (DS) also face intricate psychosocial challenges (192). Furthermore, the illness of a child may lead to a familial breakdown, and a single parent raising a child may be confronted with heightened difficulty and responsibility, thereby intensifying psychological trauma for both the parents and the child. Consequently, the prioritization of psychological aid for patients with rare diseases and their caregivers is emphasized (30, 164).

## 9 Public health policy and rare diseases

The management of rare diseases necessitates a comprehensive public health strategy that incorporates policy development, resource allocation, and the active involvement of diverse stakeholders, including healthcare providers, researchers, and patient advocacy groups (208, 209). Public health policy is vital for the adequate care and management of rare diseases, particularly in ensuring that sufficient resources are earmarked for diagnosis, treatment, and long-term care.

In recent years, international frameworks have emerged to advance the rights of individuals with rare diseases. A notable milestone was the passage of the United Nations General Assembly resolution A/RES/78/173 in 2021, which calls for heightened attention to the global challenges posed by rare diseases. This resolution encourages member states to formulate national plans for rare diseases, improve access to diagnostic tools, and foster research into treatments. Furthermore, it emphasises the crucial need for international collaboration and the sharing of information to tackle the public health challenges associated with rare diseases (210). These initiatives underscore the significance of integrating considerations for rare diseases into public health policy to ensure that individuals with these conditions are not marginalized. Furthermore, value-based healthcare models, which focus on patient outcomes and cost-effectiveness, can be instrumental in facilitating access to essential treatments, including orphan drugs (103, 211). Implementing educational initiatives designed to raise awareness of rare diseases among healthcare professionals and the general public is critical for reducing diagnostic delays and enhancing care (107).

Patient advocacy groups are essential in influencing public health policy regarding rare diseases. These organizations offer invaluable insights into patient needs, promote awareness, and advocate for policy changes that emphasize research and funding for rare diseases (212). Collaborative efforts between advocacy groups and governmental bodies can significantly improve the quality of care and support available to families affected by rare diseases.

Successful policy models in countries such as the USA and members of the European Union demonstrate that integrating rare disease management into national health systems is not only feasible but can also lead to significant improvements in patient outcomes (213–215). Recently, Latin America has introduced policies and recommendations aimed at enhancing access to equitable healthcare services for patients with rare diseases (216). These models typically involve the establishment of national registries, coordination among healthcare providers, and the formation of public-private partnerships to ensure sustainable access to care and treatment. For instance, initiatives like the European Reference Networks (ERNs) create platforms for sharing best practices and fostering collective learning among healthcare providers across Europe (217). By prioritising cooperation, countries can collaborate to promote data sharing, advance clinical research, and develop innovative treatment options that extend beyond their borders.

Investing in registry-based research can yield valuable insights into rare diseases by enabling the systematic collection of data that informs policy decisions. These registries facilitate longitudinal studies that evaluate treatment outcomes and patient experiences, thereby generating the essential evidence needed to develop informed guidelines and optimize resource allocation (218, 219). While data scarcity and healthcare disparities persist, significant opportunities exist for improvement and innovation (220). Therefore, public health policies play a crucial role in enhancing the circumstances of children with rare diseases. By focusing on accessibility, advocacy, funding, and education, public health initiatives can improve the quality of life for affected families and promote advancements in research and treatment. A coordinated global effort that prioritises rare diseases will be vital in addressing the ongoing challenges faced by this vulnerable population.

## 10 Challenges

Rare diseases pose a significant global challenge for individuals, families, and the healthcare system (Table 2) (221). The heterogeneity of epidemiological approaches and inconsistent reporting constrained the identification of high-quality population-based studies and data collection. The generalisability of results to other regions may be compromised by including solely global, European, or American data (22, 123).

**Table 2:** Challenges faced by patients and their families with rare diseases.

Domain	Description	Consequence
Medical challenges		
Delayed diagnosis	Rare diseases often present with non-specific symptoms that overlap with more common conditions, leading to delayed or incorrect diagnoses	This delay can result in prolonged suffering, disease progression, and missed opportunities for early intervention
Lack of effective treatments	Many rare diseases have limited or no effective treatments available	Patients may endure chronic symptoms and progressive disability, with few options to alleviate their condition
Complex medical management	Managing rare diseases often requires a multidisciplinary approach and specialized care	Coordination among various healthcare providers can be complex, leading to fragmented care and increased risk of complications
Access to expertise	Expertise in rare diseases is often concentrated in a few specialized centres	Patients may need to travel long distances for consultations and treatments, which can be physically and financially burdensome
Psychological and emotional challenges		
Emotional stress	Living with a rare disease can cause significant emotional and psychological stress for patients and their families	Increased risk of anxiety, depression, and feelings of isolation or helplessness
Stigma and misunderstanding	The general public and healthcare professionals often misunderstand RDs	Patients and families may experience stigma, discrimination, or lack of empathy and support
Uncertainty about the future	The unpredictable nature and progression of many rare diseases can create a sense of uncertainty	This uncertainty can make it difficult for families to plan for the future, adding to their emotional burden
Social challenges		
Social isolation	The rarity of the disease can lead to a lack of connection with others who understand the condition	Patients and families may feel isolated and lack social support networks
Educational barriers	Children with rare diseases may face challenges in educational settings, including frequent absences and the need for special accommodations	Ensuring appropriate educational support and understanding from school systems can be difficult
Impact on siblings and family dynamics	Siblings of children with rare diseases may feel neglected or burdened	Family dynamics can be strained, leading to stress and conflict within the household
Financial challenges		
High medical costs	Treatment, medications, and specialized care for rare diseases can be extremely expensive	Families may face significant out-of-pocket expenses, even with insurance coverage, leading to financial strain or debt
Lost income and employment issues	Parents or caregivers, demonstrating remarkable resilience, may need to reduce work hours or leave their jobs to care for their affected child	Loss of income can exacerbate financial difficulties and impact the family’s overall economic stability
Insurance barriers	Navigating insurance coverage for rare disease treatments can be complex and challenging, with issues such as high deductibles, limited coverage for specialized treatments, and frequent changes in coverage policies	Denials for coverage, limited access to necessary medications or therapies, and the need for frequent appeals can add to the financial and emotional stress
Advocacy and support challenges		
Lack of awareness	Public awareness of many rare diseases is low	This lack of awareness can hinder fundraising, research efforts, and policy changes that could benefit patients and families
Limited support networks	Finding support groups or communities for rare diseases can be difficult due to the small number of affected individuals	Patients and families may struggle to find others who can relate to their experiences and provide mutual support
Need for advocacy	Advocating for better healthcare policies, research funding, and support services, while demanding, holds the potential for significant positive change	Families may feel overwhelmed by the need to advocate while also managing the day-to-day challenges of living with a rare disease
Challenges in research and clinical trials		
Small patient populations	The small number of patients with any rare disease makes conducting large-scale clinical trials difficult	This can slow the development of new treatments and limit the availability of evidence-based therapies
Geographic barriers	Clinical trials and research studies are often conducted at specialized centres, which may be far from where patients live	Traveling to participate in trials can be burdensome or impractical for many families
Regulatory hurdles	Navigating the regulatory landscape for rare disease treatments can be complex	Delays in approval processes can hinder timely access to new and potentially life-saving therapies

Numerous obstacles hinder rare disease research, such as an inadequate understanding of rare diseases’ pathophysiology and natural history, along with a lack of incentives to fund the development of orphan drugs for small populations (222). Furthermore, innovative treatments may not be readily available to the general population due to factors including pricing and reimbursement decisions and the limited commercial interest of some pharmaceutical companies in investing in new orphan drug research (13, 195). An additional challenge arises in developing innovative approaches to clinical trials and health technology assessment methods in small populations, as well as the absence of aggregated primary data for estimating the reliable economic burden of rare diseases (223, 224). For instance, many African countries do not maintain national registries for rare diseases, complicating efforts to track their prevalence and fully understand the issue (225, 226). Many regions lack essential diagnostic tools, such as genomic sequencing, which can result in misdiagnoses or delays in diagnosing children with rare diseases (227, 228). Healthcare systems in numerous Latin American countries face similar challenges due to insufficient data on rare diseases, lengthy waiting times for diagnostic tests, and limited public awareness (5, 229). As a result, families often struggle to navigate the myriad medical and social challenges posed by rare diseases. Disparities in the quality of care and access to treatment for rare diseases persist across social classes and communities, even in developed economies with advanced rare disease policies, budgets, and clinical guidelines (21). Variations in the availability and accessibility of medical treatment between rural and urban areas may necessitate long-distance travel for treatment. This socio-economic divide between urban and rural areas exacerbates these challenges, as rural families have less access to medical services. There is also minimal public health education regarding rare diseases, leading to misconceptions and stigma within local communities (135, 226, 230). The development of orphan drugs for rare diseases also encounters notable challenges (231). The cost of certain available orphan drugs is prohibitively high, and many countries lack adequate insurance coverage for rare diseases, preventing families from accessing vital treatments (232).

Accessing social benefits and financial support for rare diseases has presented challenges for many individuals, particularly concerning the high costs of specialized care and treatment, especially for children diagnosed with rare diseases. Those suffering from rare diseases often experience pain, loneliness, and susceptibility to misinformation throughout the diagnosis and treatment process (36). One significant barrier to effective care in regions like Latin America is the limited access to specialized healthcare professionals (216), lack of communication between care professionals and uncoordinated appointments (233). Specialized paediatricians play a pivotal role in identifying, diagnosing, and treating children with rare diseases. It is alarming that a mere 5.3% of surveyed doctors in China were found to be knowledgeable about rare diseases (234). Similarly, a study in Australia attributed approximately 30% of diagnostic delays among families to a lack of knowledge among health professionals (167); with similar findings in Poland, where parents complained about doctors’ lack of knowledge (235). The aforementioned lack of awareness extends beyond healthcare professionals to include families, caregivers, education professionals, and peer groups, all of whom possess a limited understanding of rare diseases and their impact on the quality of life and socioeconomic burden in children (173, 236).

Therefore, comprehensive support systems are essential to address these challenges. These may encompass financial assistance, mental health services, educational support, and access to advanced therapies (Table 3). Additionally, increased research funding and policy changes are imperative to better cater to the needs of patients with rare diseases and their families, ultimately alleviating their disease burden and enhancing their overall quality of life.

**Table 3:** Recommendations for improving the diagnosis and treatment of patients with rare diseases.

Domain	Recommendation	Ref
Enhance diagnosis		
Enhance awareness	Increase awareness among healthcare providers, researchers, and the general public about RDs and their impact on patients and families	Dias et al. [134], Wright et al. [47], and Abdallah et al. [63]
Improve diagnostic criteria	Establish clear diagnostic criteria for rare diseases (RDs) to facilitate timely and accurate diagnoses	
Invest in genetic testing	Invest in genetic testing technologies to detect genetic mutations early and improve diagnosis accuracy	
Use AI-powered diagnostic tools	Leverage artificial intelligence (AI) and machine learning (ML) to develop diagnostic tools that can analyze large amounts of data and identify patterns associated with rare diseases	
Foster collaboration	Encourage collaboration between healthcare providers, researchers, and patient advocacy groups to share knowledge and best practices for diagnosing rare diseases	
Enhance the use of technology and telemedicine	Establish telehealth networks and provide training for healthcare providers on how to utilize these technologies effectively	
Promote patient registries and databases	Create centralized, secure databases that healthcare providers and researchers can access, ensuring patient consent and data privacy	
Improving treatment		
Develop targeted therapies	Develop therapies that target specific genetic mutations or biological pathways associated with RDs	Jensen et al. [82], Hoang et al. [87], and Drilon et al. [95]
Invest in orphan drug development	Encourage pharmaceutical companies to develop orphan drugs for rare diseases, which often have high unmet medical needs	
Improve access to orphan drugs	Implement policies to control drug pricing, provide subsidies or financial assistance programmes, and streamline the approval process for orphan drugs	
Improve access to treatment	Ensure that patients have access to approved treatments, including those that are not commercially available but may be available through clinical trials or compassionate use programmes	
Personalised medicine	Adopt personalised medicine approaches that take into account individual patient characteristics, such as genetic profiles, to optimize treatment outcomes	
Patient-centered care	Provide patient-centred care that focuses on the patient’s overall well-being, including emotional and psychological support and medical treatment	
Facilitate clinical trials	Simplify regulatory requirements, provide incentives for pharmaceutical companies, and create international trial networks to pool resources and patient populations	
Enhance personalised medicine approaches	Invest in technologies such as CRISPR-Cas9 and other gene-editing tools and encourage using biomarkers for targeted therapies	
Develop multidisciplinary care models	Establish centres of excellence and multidisciplinary care teams specialized in RDs, and fund the creation of these centres and ensure they are equipped with the necessary resources and trained personnel to provide comprehensive care	
Research		
Increase funding	Increase funding for RD research to accelerate the discovery of new treatments and diagnostics	Alonso Ruiz et al. [246] and Lavelle et al. [68]
Foster collaboration	Encourage collaboration between researchers, industry partners, and patient advocacy groups to share knowledge and resources	
Leverage big data	Leverage big data analytics to identify patterns and trends associated with rare diseases and accelerate research progress	
Incorporate patient input	Incorporate patient input into research design, data analysis, and interpretation to ensure that research is relevant and meaningful to patients	
Develop biomarkers	Develop biomarkers that can accurately diagnose and monitor rare diseases, enabling early intervention and personalised treatment	
Standardize data collection and sharing	Create universal data standards and ensure interoperability between different healthcare systems and databases	
Improve cross-border access to treatments	Harmonize regulatory processes and create agreements to allow patients to receive care and participate in trials internationally	
Patient and family support		
Patient education	Provide patients with accurate information about their condition, treatment options, and prognosis to empower informed decision-making	Choudhury et al. [212], Patterson et al. [7], and Depping [201]
Patient advocacy	Support patient advocacy groups that provide emotional support, education, and advocacy for patients with rare diseases	
Social support networks	Establish social support networks that connect patients with others who share similar experiences and challenges	
Mental health support	Provide mental health support services for patients and families coping with the emotional toll of living with a rare disease	
Counseling services	Offer counseling services that address the unique challenges associated with living with an RD	
Financial support mechanisms	Establish grants, subsidies, and insurance reforms to cover out-of-pocket expenses and offer financial planning services	
Care coordination	Integrate care coordination services into healthcare plans and provide training for coordinators	

By implementing these recommendations, healthcare systems can significantly enhance the diagnosis and treatment of rare diseases, thereby improving the quality of life for patients and their families.

## 11 Future research directions

Several strategies and policies have been established to enhance awareness of rare diseases and facilitate the dissemination of pertinent information within the healthcare community, particularly within the EU (166, 223). These initiatives encompass the development of rare disease registries (17, 76), active involvement of patients in research decision-making (237), application of health utility estimation techniques (238), creation of a regulatory framework and initiatives for the development of orphan drugs (127, 239), as well as the establishment of advocacy groups (240). Despite these advancements, the practical implementation of these strategies and policies remains a challenge. Therefore, it is imperative for stakeholders and policymakers to take actionable steps to implement these policies (Table 4). These actionable policy initiatives include but are not limited to:

**Table 4:** Recommendations for policymakers in improving the diagnosis and treatment of patients with rare diseases.

Policy	Recommendation	Implementation	Ref
Enhancing diagnosis and early detection			
Promote awareness and education	Develop and fund educational programmes to increase awareness of rare diseases (RDs) among healthcare professionals and the public	Integrate RD education into medical and nursing school curricula, offer continuing education credits for healthcare providers, and conduct public awareness campaigns	Martinussen et al. [18] and Huang et al. [48]
Support access to genetic testing	Ensure that genetic and genomic testing is widely accessible and affordable	Provide subsidies or coverage for genetic testing under public health insurance schemes and support the establishment of regional genetic testing centres	
Develop national rare disease registries	Establish and maintain national registries for rare diseases to collect and share data	Allocate funding for creating and maintaining these registries and ensure they are interoperable with international databases	
Improving treatment and care			
Facilitate orphan drug development and access	Implement incentives for pharmaceutical companies to develop and market orphan drugs	Offer tax credits, grant funding, market exclusivity, and expedited regulatory review for orphan drugs	Mikami [214] and Abdallah et al. [241]
Ensure comprehensive healthcare coverage	Expand healthcare coverage to include treatments, medications, and supportive services for rare diseases	Mandate insurance coverage for orphan drugs and rare disease treatments and establish public funding programmes to assist with out-of-pocket costs	
Establish centres of excellence	Designate and fund centres of excellence specialising in rare diseases	Provide grants for developing these centres, ensuring they have multidisciplinary teams and advanced diagnostic and treatment capabilities	
Supporting patients and families			
Provide financial assistance programmes	Create programmes to help families manage the financial burden of RDs	Establish grants, subsidies, and tax relief programmes for medical expenses, travel costs, and caregiver support	Choudhury and Saberwal [212] and Global Partnerships [248]
Enhance social services and support networks	Develop comprehensive support services for patients and families	Fund mental health services, respite care, and social work support to facilitate the creation of support groups and networks for rare disease communities	
Improve educational support for children	Ensure that children with RDs receive appropriate educational accommodation and support	Develop policies that mandate individualized education plans (IEPs) and provide funding for special education services and school-based healthcare providers	
Encouraging research and innovation			
Increase research funding	Allocate more public funds to rare disease research	Create dedicated research grants and fellowships and encourage public-private partnerships to support innovative research projects	Alonso et al. [246] and Patterson et al. [7]
Support clinical trials	Facilitate the development and conduct of clinical trials for RDs	Streamline regulatory processes, provide financial incentives for trial sponsors, and support the establishment of international trial networks to increase patient recruitment	
Foster collaboration and data sharing	Promote collaboration among researchers, healthcare providers, and patients	Fund and develop data-sharing platforms, establish consortia and research networks, and ensure data standardization and interoperability	
Strengthening policy and legislation			
Develop comprehensive national rare	Formulate and implement national strategies to address rare diseases	Involve stakeholders in the development of these plans, set clear goals and objectives, and allocate appropriate resources for implementation	Lopes-Júnior et al. [209] and Montserrat and Taruscio [215]
Disease plans			
Ensure regulatory flexibility	Adapt regulatory frameworks to facilitate the approval and availability of orphan drugs and treatments	Implement accelerated approval pathways, adaptive licensing, and conditional approvals for promising therapies	
Protect patient rights and access	Enact laws that protect patients’ rights with rare diseases and ensure their access to necessary care	Ensure anti-discrimination protections, mandate coverage for essential treatments, and support policies that enhance patient access to care and information	
Enhancing global collaboration			
Promote international cooperation	Foster international partnerships and collaborations to address RDs	Participate in global initiatives, share data and research findings, and harmonize regulatory standards to facilitate cross-border healthcare access	Global Partnerships and Seifert [247]
Support global research networks	Encourage the development of global research networks focused on rare diseases	Provide funding for international collaborative research projects and support platforms for global data sharing and coordination	
Facilitate access to global treatments	Ensure that patients can access treatments and clinical trials in other countries	Harmonize regulatory approvals, create agreements for cross-border healthcare, and support patients in navigating international treatment options	
Monitoring and evaluation			
Track and evaluate policy impact	Regularly monitor and evaluate the effectiveness of policies and programmes aimed at rare diseases	Establish metrics and benchmarks for success, conduct periodic reviews, and adjust policies based on feedback and outcomes	Economist Impact – Perspectives [232] and Chung et al. [213]
Engage stakeholders in policy development	Involve patients, families, healthcare providers, researchers, and advocacy groups in policy development and evaluation	Create advisory committees and forums for stakeholder input and ensure their active participation in decision-making processes	

### 11.1 Multidisciplinary care models

Governments and healthcare providers need to prioritize the implementation of multidisciplinary care models. These models should integrate the expertise of specialists, general practitioners, psychologists, social workers, and various other healthcare professionals to offer comprehensive care. Evidence has shown that such coordinated approaches enhance patient outcomes by addressing the physical, emotional, and social needs of those living with rare diseases (7, 241). For example, the Netherlands has successfully adopted a multidisciplinary strategy through the Dutch Rare Disease Registry, significantly improving patients’ quality of life and their access to vital treatments (242). The registry is not only significant for local healthcare systems but also plays a crucial role in broader initiatives across Europe aimed at gathering data on rare diseases. This integration enables stakeholders to make well-informed decisions. By prioritising standardized data collection and sharing, the registry strengthens the reliability of research efforts and the treatment protocols derived from the gathered information (243).

### 11.2 Expanding access to orphan drugs

A significant challenge in the treatment of rare diseases lies in the high cost and limited availability of orphan drugs. Policymakers should focus on developing strategies to improve access to these essential medications, particularly in resource-constrained environments. One potential approach is the establishment of public-private partnerships involving governments, pharmaceutical companies, and non-profit organizations to share the financial burden associated with orphan drug development. Furthermore, it is crucial to create frameworks for price negotiation that would make orphan drugs more affordable, especially for low- and middle-income countries (244–246).

### 11.3 Global cooperation and data sharing

There is an urgent need for enhanced international collaboration in the research and treatment of rare diseases. By developing global data-sharing platforms, countries can pool their resources, conduct larger-scale studies, improve diagnostic capabilities, and accelerate the development of orphan drugs (212, 247, 248). The success of initiatives like the European Reference Networks (ERNs), which aim to improve healthcare for rare disease patients across Europe, should serve as a model for global implementation (217). Each European Reference Network (ERN) consists of a minimum of ten healthcare providers from at least eight different member states, ensuring a diverse pool of expertise and resources (249). Beyond their role in patient care, ERNs are instrumental in advancing research related to rare diseases. By pooling clinical data and sharing findings, they contribute to the establishment of registries that enhance our understanding of disease trajectories, treatment efficacy, and patient outcomes. This collaborative research is crucial, as it provides the evidence necessary to develop new therapies and improve standards of care (250). Embracing ideas such as the Findable, Accessible, Interoperable, and Reusable (FAIR) principles could facilitate better cross-border collaboration and data sharing, fostering innovative research and application of solutions in clinical settings (251).

The practical implementation of these measures will address the immediate and long-term needs of those affected and contribute to sustained progress in rare disease care and research. Furthermore, future research endeavors should prioritize specific areas to gain a deeper understanding of the true impact of rare diseases on the quality of life in paediatric patients, as detailed in Table 5. By focusing on these prospective research directions, the scientific community can advance its knowledge, enhance clinical outcomes, and improve the lives of patients and families impacted by rare diseases. Collaborative efforts transcending disciplines and borders will be indispensable in overcoming the complexities and substantial challenges associated with these conditions.

**Table 5:** Future research directions.

Domain	Direction	Ref
Genetics and genomics		
Identification of novel genetic variants	Explore whole-genome sequencing and advanced genomic technologies to identify new genetic variants associated with RDs; utilize population-scale sequencing projects to uncover rare variants and their implications for disease pathology	Baynam et al. [227] and Hou et al. [49]
Functional genomics and pathways	Investigate functional genomics approaches to elucidate disease mechanisms and pathways underlying rare diseases; utilize CRISPR-Cas9 and other gene-editing techniques to study gene function and validate potential therapeutic targets	
Epigenetics and gene regulation	Study epigenetic modifications and non-coding RNA regulation in rare diseases to understand how these mechanisms influence disease onset and progression; explore the role of chromatin remodeling and transcriptional regulation in rare disease pathogenesis	
Disease mechanisms and pathophysiology		
Mechanistic studies metabolic and biochemical pathways	Conduct in-depth mechanistic studies to uncover disease-specific pathways, cellular mechanisms, and biomarkers; utilize advanced imaging techniques and organoid models to mimic disease conditions and study tissue-specific effects Investigate metabolic and biochemical abnormalities in RDs to identify metabolic signatures and potential therapeutic targets; Explore the role of mitochondrial dysfunction, metabolic reprogramming, and nutrient-sensing pathways	Kolb et al. [51]
Immune system dysregulation	Study immune system dysregulation in rare diseases, including autoimmunity, inflammation, and immunodeficiency; Investigate the interplay between genetic mutations, immune cell dysfunction, and disease manifestation	
Therapeutic approaches and drug development		
Precision medicine and targeted therapies	Develop personalised treatment strategies based on genetic and molecular profiles of patients with rare diseases; Implement pharmacogenomics approaches to predict drug responses and optimize treatment outcomes	Frangoul et al. [78] and Papaioannou et al. [81]
Gene therapy and genome editing	Advanced gene therapy techniques, including viral vectors and genome editing tools, for correcting genetic defects underlying RDs; Explore novel delivery systems and improve the safety and efficacy profiles of gene therapies	
Drug repurposing and screening	Explore drug repurposing opportunities for rare diseases by screening existing compounds for new therapeutic indications; Develop high-throughput screening platforms and computational approaches to identify potential drug candidates	
Data sharing and collaborative networks		
Data integration and analytics global collaborations and networks	Develop integrated data-sharing and analysis platforms to facilitate collaborative research efforts across institutions and countries; Implement artificial intelligence and machine learning algorithms to analyze large-scale genomic, clinical, and phenotypic data Establish international consortia and networks to share resources, data, and expertise in rare disease research; to accelerate discoveries, foster collaborations between researchers, clinicians, patient advocacy groups, and industry stakeholders	Hedley et al. [250] and Global Partnerships [248]
Patient-centred research and outcomes		
Patient-reported outcomes	Incorporate patient-reported outcomes and quality-of-life measures into rare disease research studies; develop standardized tools for assessing patient outcomes, treatment efficacy, and long-term prognosis	Seifert [247] and Khosla and Valdez [208]
Natural history studies	Conduct longitudinal studies and natural history assessments to characterize disease progression and variability in rare diseases; identify predictive biomarkers and disease milestones to guide clinical management and intervention strategies	
Policy, advocacy, and healthcare systems		
Healthcare access and policy	Advocate for policies that improve access to healthcare services, orphan drugs, and specialized care for patients with rare diseases; address regulatory challenges and promote incentives for orphan drug development and market access	Alonso et al. [246] and Scherman Fétro [245]
Rare disease awareness and education	Increase public and healthcare provider awareness of rare diseases through education campaigns and professional training; support initiatives that promote early diagnosis, patient empowerment, and community engagement	

## 12 Conclusion

Rare diseases present a considerable global burden, impacting millions of individuals worldwide, with children constituting the most affected demographic. Inadequacies in disease prevalence estimates underscore significant gaps in our understanding of the epidemiology of rare diseases, particularly within the paediatric cohort. The persistent nature of numerous rare diseases imposes substantial physical, emotional, and social burdens on affected children and their families, profoundly affecting their quality of life. These challenges are further exacerbated by the absence of suitable and accessible treatments for various rare diseases, resulting in numerous children remaining undiagnosed or receiving suboptimal care. The profound ramifications for the quality of life of affected children and their families underscore the imperative to develop efficacious treatments and enhance healthcare accessibility to alleviate the disproportionate burden imposed by these conditions.

## References

[1] Abozaid GM Kerr K McKnight A Al-Omar HA. Criteria to define rare diseases and orphan drugs: a systematic review protocol. BMJ Open. (2022) 12:e062126. doi: 10.1136/bmjopen-2022-062126, PMID: 35906057 PMC9345065

[2] Raycheva R Kostadinov K Mitova E Bogoeva N Iskrov G Stefanov G. Challenges in mapping European rare disease databases, relevant for ML-based screening technologies in terms of organizational, FAIR and legal principles: scoping review. Front Public Health. (2023) 11:1214766. doi: 10.3389/fpubh.2023.1214766, PMID: 37780450 PMC10540868

[3] Vicente E Pruneda L Ardanaz E. Regarding the estimations of people affected by rare diseases. Eur J Hum Genet. (2021) 29:1032–3. doi: 10.1038/s41431-020-00763-z, PMID: 33262443 PMC8187376

[4] Cheung RY Cohen JC Illingworth P. Orphan drug policies: implications for the United States, Canada, and developing countries. Health Law J. (2004) 12:183–200. PMID: 16539081

[5] Giugliani R Castillo Taucher S Hafez S Oliveira JB Rico-Restrepo M Rozenfeld P. Opportunities and challenges for newborn screening and early diagnosis of rare diseases in Latin America. Front Genet. (2022) 13:1053559. doi: 10.3389/fgene.2022.1053559 36568372 PMC9773081

[6] Rare Diseases International. (2025). Vision & Mission. Available online at: https://www.rarediseasesinternational.org/vision-mission/ (Accessed January 18, 2025).

[7] Patterson AM O’Boyle M VanNoy GE Dies KA. Emerging roles and opportunities for rare disease patient advocacy groups. Ther Adv Rare Dis. (2023) 4:26330040231164425. doi: 10.1177/26330040231164425, PMID: 37197559 PMC10184204

[8] Marwaha S Knowles JW Ashley EA. A guide for the diagnosis of rare and undiagnosed disease: beyond the exome. Genome Med. (2022) 14:23. doi: 10.1186/s13073-022-01026-w, PMID: 35220969 PMC8883622

[9] Schaaf J Sedlmayr M Schaefer J Storf H. Diagnosis of rare diseases: a scoping review of clinical decision support systems. Orphanet J Rare Dis. (2020) 15:263. doi: 10.1186/s13023-020-01536-z, PMID: 32972444 PMC7513302

[10] D’Angelo CS Hermes A McMaster CR Prichep E Richer É Van Der Westhuizen FH. Barriers and considerations for diagnosing rare diseases in indigenous populations. Front Pediatr. (2020) 8:579924. doi: 10.3389/fped.2020.579924, PMID: 33381478 PMC7767925

[11] Hsu JC Wu HC Feng WC Chou CH Lai ECC Lu CY. Disease and economic burden for rare diseases in Taiwan: a longitudinal study using Taiwan’s National Health Insurance Research Database. PLoS One. (2018) 13:e0204206. doi: 10.1371/journal.pone.0204206 30240449 PMC6150502

[12] Tsitsani P Katsaras G Soteriades ES. Barriers to and facilitators of providing Care for Adolescents Suffering from rare diseases: a mixed systematic review. Pediatr Rep. (2023) 15:462–82. doi: 10.3390/pediatric15030043, PMID: 37606447 PMC10443320

[13] Klimova B Storek M Valis M Kuca K. Global view on rare diseases: a Mini review. Curr Med Chem. (2017) 24:3153–3158. doi: 10.2174/0929867324666170511111803 28494745

[14] Somanadhan S O’Donnell R Bracken S McNulty S Sweeney A O’Toole D. Children and young people’s experiences of living with rare diseases: an integrative review. J Pediatr Nurs. (2023) 68:e16–26. doi: 10.1016/j.pedn.2022.10.014, PMID: 36443134

[15] Boettcher J Boettcher M Wiegand-Grefe S Zapf H. Being the pillar for children with rare diseases-a systematic review on parental quality of life. Int J Environ Res Public Health. (2021) 18:4993. doi: 10.3390/ijerph18094993, PMID: 34066738 PMC8125857

[16] Mingorance A. Drivers of orphan drug development. ACS Med Chem Lett. (2018) 9:962–4. doi: 10.1021/acsmedchemlett.8b00438, PMID: 30344899 PMC6187403

[17] Ruseckaite R Mudunna C Caruso M Helwani F Millis N Lacaze P. Current state of rare disease registries and databases in Australia: a scoping review. Orphanet J Rare Dis. (2023) 18:216. doi: 10.1186/s13023-023-02823-1, PMID: 37501152 PMC10373259

[18] Martinussen J Chalk M Elliott J Gallacher L. Receiving genomic sequencing results through the Victorian undiagnosed disease program: exploring parental experiences. J Pers Med. (2022) 12:1250. doi: 10.3390/jpm12081250, PMID: 36013198 PMC9410238

[19] Liu J Yu Y Zhong M Ma C Shao R. Long way to go: Progress of orphan drug accessibility in China from 2017 to 2022. Front Pharmacol. (2023) 14:1138996. doi: 10.3389/fphar.2023.1138996, PMID: 36969835 PMC10031016

[20] Guo J Liu P Chen L Lv H Li J Yu W. National Rare Diseases Registry System (NRDRS): China’s first nation-wide rare diseases demographic analyses. Orphanet J Rare Dis. (2021) 16:515. doi: 10.1186/s13023-021-02130-7, PMID: 34922584 PMC8684272

[21] Adachi T El-Hattab AW Jain R Nogales Crespo KA Quirland Lazo CI Scarpa M. Enhancing equitable access to rare disease diagnosis and treatment around the world: a review of evidence, policies, and challenges. Int J Environ Res Public Health. (2023) 20:4732. doi: 10.3390/ijerph20064732, PMID: 36981643 PMC10049067

[22] Nguengang Wakap S Lambert DM Olry A Rodwell C Gueydan C Lanneau V. Estimating cumulative point prevalence of rare diseases: analysis of the Orphanet database. Eur J Hum Genet EJHG. (2020) 28:165–73. doi: 10.1038/s41431-019-0508-0, PMID: 31527858 PMC6974615

[23] Bush A Chitty L Harcourt J Hewitt RJ Nicholson AG. Congenital lung disease In: R. W. Wilmott, R. Deterding, A. Li, F. Ratjen, P. Sly, H. J. Zar et al. (Eds). Kendig’s Disorders of the Respiratory Tract in Children (*9th* Edn), Elsevier (2019). 289–337.e8. doi: 10.1016/B978-0-323-44887-1.00018-3

[24] Rafeeq MM Murad HAS. Cystic fibrosis: current therapeutic targets and future approaches. J Transl Med. (2017) 15:84. doi: 10.1186/s12967-017-1193-9, PMID: 28449677 PMC5408469

[25] Orso M Migliore A Polistena B Russo E Gatto F Monterubbianesi M. Duchenne muscular dystrophy in Italy: a systematic review of epidemiology, quality of life, treatment adherence, and economic impact. PLoS One. (2023) 18:e0287774. doi: 10.1371/journal.pone.0287774, PMID: 37368924 PMC10298760

[26] Kozel BA Barak B Ae Kim C Mervis CB Osborne LR Porter M. Williams syndrome. Nat Rev Dis Primer. (2021) 7:42. doi: 10.1038/s41572-021-00276-z, PMID: 34140529 PMC9437774

[27] Cheema HA Waheed N Saeed A. Unusual case of juvenile Tay-Sachs disease. BMJ Case Rep. (2019) 12:e230140. doi: 10.1136/bcr-2019-230140, PMID: 31519716 PMC6747916

[28] Vidal S Xiol C Pascual-Alonso A O’Callaghan M Pineda M Armstrong J. Genetic landscape of Rett syndrome Spectrum: improvements and challenges. Int J Mol Sci. (2019) 20:3925. doi: 10.3390/ijms20163925, PMID: 31409060 PMC6719047

[29] Gonzalo S Kreienkamp R Askjaer P. Hutchinson-Gilford progeria syndrome: a premature aging disease caused by LMNA gene mutations. Ageing Res Rev. (2017) 33:18–29. doi: 10.1016/j.arr.2016.06.007, PMID: 27374873 PMC5195863

[30] Borski K. Characteristics of rare diseases. Gen Intern Med Clin Innov. (2017) 2:3–4. doi: 10.15761/GIMCI.1000142

[31] Tripathy K Nanda T. The influence of environmental and genetic factors on various disorders and diseases. J Genet Syndr Gene Ther. (2011) 2. doi: 10.4172/2157-7412.S11-001

[32] Ren ZM Li WJ Xing ZH Fu XY Zhang JY Chen YS. Detecting rare thalassemia in children with anemia using third-generation sequencing. Hematol Amst Neth. (2023) 28:2241226. doi: 10.1080/16078454.2023.2241226, PMID: 37548329

[33] Tukker AM Royal CD Bowman AB McAllister KA. The impact of environmental factors on monogenic Mendelian diseases. Toxicol Sci Off J Soc Toxicol. (2021) 181:3–12. doi: 10.1093/toxsci/kfab022, PMID: 33677604 PMC8599782

[34] Lazareva TE Barbitoff YA Nasykhova YA Pavlova NS Bogaychuk PM Glotov AS. Statistical dissection of the genetic determinants of phenotypic heterogeneity in genes with multiple associated rare diseases. Genes. (2023) 14:2100. doi: 10.3390/genes14112100, PMID: 38003043 PMC10671084

[35] Zhu CY Hong W Wang L Ding LJ Zhang X. Department of health sciences, National Natural Science Foundation of China, Beijing 100085, China, et al. towards key scientific questions in the diagnosis and treatment of rare diseases: summary from the 297th meeting of the Shuangqing forum. Zool Res. (2022) 43:234–6. doi: 10.24272/j.issn.2095-8137.2022.068, PMID: 35194981 PMC8920843

[36] Tirado Perez IS Lopez SP Zarate Vergara AC. Rare diseases: a current view. J Pediatr Care. (2017) 3. doi: 10.21767/2471-805X.100031

[37] Rillig F Grüters A Schramm C Krude H. The interdisciplinary diagnosis of rare diseases. Dtsch Arzteblatt Int. (2022) 119:469–75. doi: 10.3238/arztebl.m2022.0219 PMC9664985 35635437

[38] Austin CP Cutillo CM Lau LPL Jonker AH Rath A Julkowska D. Future of rare diseases research 2017-2027: an IRDiRC perspective. Clin Transl Sci. (2018) 11:21–7. doi: 10.1111/cts.12500, PMID: 28796445 PMC5759721

[39] Taruscio D Floridia G Salvatore M Groft SC Gahl WA. Undiagnosed diseases: Italy-US collaboration and international efforts to tackle rare and common diseases lacking a diagnosis In: Posada De La Paz M Taruscio D Groft SC, editors. Rare diseases epidemiology: Update and overview. Cham: Springer International Publishing (2017). 25–38. 10.1007/978-3-319-67144-4_2 29214564

[40] 100,000 Genomes Project Pilot Investigators Smedley D Smith KR Martin A Thomas EA McDonagh EM. 100,000 genomes pilot on rare-disease diagnosis in health care — preliminary report. N Engl J Med. (2021) 385:1868–80. doi: 10.1056/NEJMoa2035790, PMID: 34758253 PMC7613219

[41] Licata L Via A Turina P Babbi G Benevenuta S Carta C. Resources and tools for rare disease variant interpretation. Front Mol Biosci. (2023) 10:1169109. doi: 10.3389/fmolb.2023.1169109, PMID: 37234922 PMC10206239

[42] Bavisetty S Grody WW Yazdani S. Emergence of pediatric rare diseases: review of present policies and opportunities for improvement. Rare Dis. (2013) 1:e23579. doi: 10.4161/rdis.23579, PMID: 25002987 PMC3932940

[43] Faye F Crocione C Anido de Peña R Bellagambi S Escati Peñaloza L Hunter A. Time to diagnosis and determinants of diagnostic delays of people living with a rare disease: results of a rare barometer retrospective patient survey. Eur J Hum Genet EJHG. (2024) 32:1116–26. doi: 10.1038/s41431-024-01604-z, PMID: 38755315 PMC11369105

[44] Maver A Lohmann K Borovečki F Wolstenholme N Taylor RL Spielmann M. Quality assurance for next-generation sequencing diagnostics of rare neurological diseases in the European reference network. Eur J Hum Genet EJHG. (2024) 32:1014–21. doi: 10.1038/s41431-024-01639-2, PMID: 38839988 PMC11292006

[45] Frésard L Montgomery SB. Diagnosing rare diseases after the exome. Mol Case Stud. (2018) 4:a003392. doi: 10.1101/mcs.a003392, PMID: 30559314 PMC6318767

[46] Fu MP Merrill SM Sharma M Gibson WT Turvey SE Kobor MS. Rare diseases of epigenetic origin: challenges and opportunities. Front Genet. (2023) 14:1113086. doi: 10.3389/fgene.2023.1113086 36814905 PMC9939656

[47] Wright CF FitzPatrick DR Firth HV. Paediatric genomics: diagnosing rare disease in children. Nat Rev Genet. (2018) 19:253–68. doi: 10.1038/nrg.2017.116, PMID: 29398702

[48] Huang X Wu D Zhu L Wang W Yang R Yang J. Application of a next-generation sequencing (NGS) panel in newborn screening efficiently identifies inborn disorders of neonates. Orphanet J Rare Dis. (2022) 17:66. doi: 10.1186/s13023-022-02231-x, PMID: 35193651 PMC8862216

[49] Hou YCC Yu HC Martin R Cirulli ET Schenker-Ahmed NM Hicks M. Precision medicine integrating whole-genome sequencing, comprehensive metabolomics, and advanced imaging. Proc Natl Acad Sci USA. (2020) 117:3053–62. doi: 10.1073/pnas.1909378117, PMID: 31980526 PMC7022190

[50] Pawliński Ł Tobór E Suski M Biela M Polus A Kieć-Wilk B. Proteomic biomarkers in Gaucher disease. J Clin Pathol. (2021) 74:25–9. doi: 10.1136/jclinpath-2020-206580, PMID: 32409598

[51] Kolb SJ Coffey CS Yankey JW Krosschell K Arnold WD Rutkove SB. Natural history of infantile-onset spinal muscular atrophy. Ann Neurol. (2017) 82:883–91. doi: 10.1002/ana.25101, PMID: 29149772 PMC5776712

[52] Bax BE. Biomarkers in rare diseases. Int J Mol Sci. (2021) 22:673. doi: 10.3390/ijms22020673, PMID: 33445477 PMC7827027

[53] Ferlini A Gross ES Garnier N. Rare diseases’ genetic newborn screening as the gateway to future genomic medicine: the Screen4Care EU-IMI project. Orphanet J Rare Dis. (2023) 4:310. doi: 10.1186/s13023-023-02916-x PMC10548672 37794437

[54] Zhao H Du C Yang G Wang Y. Diagnosis, treatment, and research status of rare diseases related to birth defects. Intractable Rare Dis Res. (2023) 12:148–60. doi: 10.5582/irdr.2023.01052, PMID: 37662624 PMC10468410

[55] Martin S Angolini E Audi J Bertini E Bruno LP Coulter J. Patient preferences in genetic newborn screening for rare diseases: study protocol. BMJ Open. (2024) 14:e081835. doi: 10.1136/bmjopen-2023-081835, PMID: 38643010 PMC11056621

[56] Wong CS Kogon AJ Warady BA Furth SL Lantos JD Wilfond BS. Ethical and policy considerations for genomic testing in pediatric research: the path toward disclosing individual research results. Am J Kidney Dis Off J Natl Kidney Found. (2019) 73:837–45. doi: 10.1053/j.ajkd.2019.01.020, PMID: 30879919 PMC6548468

[57] Kilkku N Halkoaho A. Informed consent, genomic research and mental health: a integrative review. Nurs Ethics. (2022) 29:973–87. doi: 10.1177/09697330211066573, PMID: 35119339 PMC9289972

[58] Kruse J Mueller R Aghdassi AA Lerch MM Salloch S. Genetic testing for rare diseases: a systematic review of ethical aspects. Front Genet. (2021) 12:701988. doi: 10.3389/fgene.2021.701988, PMID: 35154238 PMC8826556

[59] Rodriguez-Monguio R Spargo T Seoane-Vazquez E. Ethical imperatives of timely access to orphan drugs: is possible to reconcile economic incentives and patients’ health needs? Orphanet J Rare Dis. (2017) 12:1. doi: 10.1186/s13023-016-0551-7, PMID: 28057032 PMC5217554

[60] Brett GR Martyn M Lynch F de Silva MG Ayres S Gallacher L. Parental experiences of ultrarapid genomic testing for their critically unwell infants and children. Genet Med. (2020) 22:1976–85. doi: 10.1038/s41436-020-0912-4, PMID: 32719395

[61] Chediak L Bedlington N Gadson A Kent A Khalek AA Rosen L. Unlocking sociocultural and community factors for the global adoption of genomic medicine. Orphanet J Rare Dis. (2022) 17:191. doi: 10.1186/s13023-022-02328-3, PMID: 35549752 PMC9097338

[62] Lewis C Sanderson S Hill M Patch C Searle B Hunter A. Parents’ motivations, concerns and understanding of genome sequencing: a qualitative interview study. Eur J Hum Genet. (2020) 28:874–84. doi: 10.1038/s41431-020-0575-2, PMID: 32001839 PMC7316711

[63] Abdallah S Sharifa M Almadhoun MK Khawar MM Shaikh U Balabel KM. The impact of artificial intelligence on optimizing diagnosis and treatment plans for rare genetic disorders. Cureus. 15:e46860 37954711 10.7759/cureus.46860 PMC10636514

[64] Wojtara M Rana E Rahman T Khanna P Singh H. Artificial intelligence in rare disease diagnosis and treatment. Clin Transl Sci. (2023) 16:2106–11. doi: 10.1111/cts.13619, PMID: 37646577 PMC10651639

[65] Schaaf J Prokosch HU Boeker M Schaefer J Vasseur J Storf H. Interviews with experts in rare diseases for the development of clinical decision support system software - a qualitative study. BMC Med Inform Decis Mak. (2020) 20:230. doi: 10.1186/s12911-020-01254-3, PMID: 32938448 PMC7493382

[66] Schaaf J Sedlmayr M Sedlmayr B Prokosch HU Storf H. Evaluation of a clinical decision support system for rare diseases: a qualitative study. BMC Med Inform Decis Mak. (2021) 21:65. doi: 10.1186/s12911-021-01435-8, PMID: 33602191 PMC7890997

[67] Ferket BS Baldwin Z Murali P Pai A Mittendorf KF Russell HV. Cost-effectiveness frameworks for comparing genome and exome sequencing versus conventional diagnostic pathways: a scoping review and recommended methods. Genet Med Off J Am Coll Med Genet. (2022) 24:2014–27. doi: 10.1016/j.gim.2022.06.004, PMID: 35833928 PMC9997042

[68] Lavelle TA Feng X Keisler M Cohen JT Neumann PJ Prichard D. Cost-effectiveness of exome and genome sequencing for children with rare and undiagnosed conditions. Genet Med Off J Am Coll Med Genet. (2022) 24:1349–61. doi: 10.1016/j.gim.2022.03.005, PMID: 35396982

[69] Chassagne A Pélissier A Houdayer F Cretin E Gautier E Salvi D. Exome sequencing in clinical settings: preferences and experiences of parents of children with rare diseases (SEQUAPRE study). Eur J Hum Genet EJHG. (2019) 27:701–10. doi: 10.1038/s41431-018-0332-y, PMID: 30710147 PMC6461801

[70] Elliott AM. Genetic counseling and genome sequencing in pediatric rare disease. Cold Spring Harb Perspect Med. (2020) 10:a036632. doi: 10.1101/cshperspect.a036632, PMID: 31501267 PMC7050583

[71] Crellin E Martyn M McClaren B Gaff C. What matters to parents? A scoping review of parents’ service experiences and needs regarding genetic testing for rare diseases. Eur J Hum Genet EJHG. (2023) 31:869–78. doi: 10.1038/s41431-023-01376-y, PMID: 37308600 PMC10400618

[72] Qiao L Liu X Shang J Zuo W Xu T Qu J. Evaluating the national system for rare diseases in China from the point of drug access: progress and challenges. Orphanet J Rare Dis. (2022) 17:352. doi: 10.1186/s13023-022-02507-2, PMID: 36088349 PMC9463840

[73] Narayanan S Mainz JG Gala S Tabori H Grossoehme D. Adherence to therapies in cystic fibrosis: a targeted literature review. Expert Rev Respir Med. (2017) 11:129–45. doi: 10.1080/17476348.2017.1280399, PMID: 28107795

[74] Groft SC Posada M Taruscio D. Progress, challenges and global approaches to rare diseases. Acta Paediatr. (2021) 110:2711–6. doi: 10.1111/apa.15974, PMID: 34105798

[75] Cortial L Nguyen C Julkowska D Cocqueel-Tiran F Moliner AM Blin O. Managing rare diseases: examples of national approaches in Europe, North America and East Asia. Rare Dis Orphan Drugs J. (2022) 1:10. doi: 10.20517/rdodj.2022.02

[76] Jansen-van der Weide MC Gaasterland CMW Roes KCB Pontes C Vives R Sancho A. Rare disease registries: potential applications towards impact on development of new drug treatments. Orphanet J Rare Dis. (2018) 13. doi: 10.1186/s13023-018-0836-0, PMID: 30185208 PMC6126025

[77] Dabbous O Chachoua L Aballéa S Sivignon M Persson U Petrou S. Valuation of treatments for rare diseases: a systematic literature review of societal preference studies. Adv Ther. (2023) 40:393–424. doi: 10.1007/s12325-022-02359-z, PMID: 36451072 PMC9898379

[78] Frangoul H Altshuler D Cappellini MD Chen YS Domm J Eustace BK. CRISPR-Cas9 gene editing for sickle cell disease and β-thalassemia. N Engl J Med. (2021) 384:252–60. doi: 10.1056/NEJMoa2031054, PMID: 33283989

[79] Gillmore JD Gane E Taubel J Kao J Fontana M Maitland ML. CRISPR-Cas9 in Vivo gene editing for transthyretin amyloidosis. N Engl J Med. (2021) 385:493–502. doi: 10.1056/NEJMoa2107454, PMID: 34215024

[80] Pavan E Ormazabal M Peruzzo P Vaena E Rozenfeld P Dardis A. CRISPR/Cas9 editing for Gaucher disease modelling. Int J Mol Sci. (2020) 21:3268. doi: 10.3390/ijms21093268, PMID: 32380730 PMC7246564

[81] Papaioannou I Owen JS Yáñez-Muñoz RJ. Clinical applications of gene therapy for rare diseases: a review. Int J Exp Pathol. (2023) 104:154–76. doi: 10.1111/iep.12478, PMID: 37177842 PMC10349259

[82] Jensen TL Gøtzsche CR Woldbye DPD. Current and future prospects for gene therapy for rare genetic diseases affecting the brain and spinal cord. Front Mol Neurosci. (2021) 14:695937. doi: 10.3389/fnmol.2021.695937, PMID: 34690692 PMC8527017

[83] Zogg H Singh R Ro S. Current advances in RNA therapeutics for human diseases. Int J Mol Sci. (2022) 23:2736. doi: 10.3390/ijms23052736, PMID: 35269876 PMC8911101

[84] Mercuri E Darras BT Chiriboga CA Day JW Campbell C Connolly AM. Nusinersen versus sham control in later-onset spinal muscular atrophy. N Engl J Med. (2018) 378:625–35. doi: 10.1056/NEJMoa1710504, PMID: 29443664

[85] Adams D Gonzalez-Duarte A O’Riordan WD Yang CC Ueda M Kristen AV. Patisiran, an RNAi therapeutic, for hereditary transthyretin amyloidosis. N Engl J Med. (2018) 379:11–21. doi: 10.1056/NEJMoa1716153, PMID: 29972753

[86] Aghajanian H Rurik JG Epstein JA. CAR-based therapies: opportunities for immuno-medicine beyond cancer. Nat Metab. (2022) 4:163–9. doi: 10.1038/s42255-022-00537-5, PMID: 35228742 PMC9947862

[87] Hoang DM Pham PT Bach TQ Ngo ATL Nguyen QT Phan TTK. Stem cell-based therapy for human diseases. Signal Transduct Target Ther. (2022) 7:1–41. doi: 10.1038/s41392-022-01134-4, PMID: 35933430 PMC9357075

[88] Motte J Sgodzai M Schneider-Gold C Steckel N Mika T Hegelmaier T. Treatment of concomitant myasthenia gravis and Lambert-Eaton myasthenic syndrome with autologous CD19-targeted CAR T cells. Neuron. (2024) 112:1757–1763.e2. doi: 10.1016/j.neuron.2024.04.014, PMID: 38697115

[89] Staedtke V Anstett K Bedwell D Giovannini M Keeling K Kesterson R. Gene-targeted therapy for neurofibromatosis and schwannomatosis: the path to clinical trials. Clin Trials Lond Engl. (2024) 21:51–66. doi: 10.1177/17407745231207970, PMID: 37937606

[90] De Boeck K Amaral MD. Progress in therapies for cystic fibrosis. Lancet Respir Med. (2016) 4:662–74. doi: 10.1016/S2213-2600(16)00023-0 27053340

[91] Jameson E Jones S Remmington T. Enzyme replacement therapy with laronidase (Aldurazyme®) for treating mucopolysaccharidosis type I. Cochrane Database Syst Rev. (2019) 2021. doi: 10.1002/14651858.CD009354.pub5, PMID: 31211405 PMC6581069

[92] Sims KB Pastores GM Weinreb NJ Barranger J Rosenbloom BE Packman S. Improvement of bone disease by imiglucerase (Cerezyme) therapy in patients with skeletal manifestations of type 1 Gaucher disease: results of a 48-month longitudinal cohort study. Clin Genet. (2008) 73:430–40. doi: 10.1111/j.1399-0004.2008.00978.x, PMID: 18312448 PMC2440418

[93] Ditters IAM van der Beek NAME Brusse E van der Ploeg AT van den Hout JMP Huidekoper HH. Home-based enzyme replacement therapy in children and adults with Pompe disease; a prospective study. Orphanet J Rare Dis. (2023) 18:108. doi: 10.1186/s13023-023-02715-4, PMID: 37158969 PMC10169363

[94] De Vivo DC Bertini E Swoboda KJ Hwu WL Crawford TO Finkel RS. Nusinersen initiated in infants during the presymptomatic stage of spinal muscular atrophy: interim efficacy and safety results from the phase 2 NURTURE study. Neuromuscul Disord NMD. (2019) 29:842–56. doi: 10.1016/j.nmd.2019.09.007, PMID: 31704158 PMC7127286

[95] Drilon A Laetsch TW Kummar S DuBois SG Lassen UN Demetri GD. Efficacy of Larotrectinib in TRK fusion-positive cancers in adults and children. N Engl J Med. (2018) 378:731–9. doi: 10.1056/NEJMoa1714448, PMID: 29466156 PMC5857389

[96] van Eeghen AM Bruining H Wolf NI Bergen AA Houtkooper RH van Haelst MM. Personalized medicine for rare neurogenetic disorders: can we make it happen? Cold Spring Harb Mol Case Stud. (2022) 8:a006200. doi: 10.1101/mcs.a006200 35332073 PMC8958924

[97] Scarpa M Ceci A Tomanin R Mincarone P Begley D. Personalised medicine in paediatrics: individualising treatment in children with rare neurological diseases. EPMA J. (2011) 2:231–9. doi: 10.1007/s13167-011-0081-2, PMID: 23199151 PMC3405387

[98] Kim J Hu C Moufawad El Achkar C Black LE Douville J Larson A. Patient-customized oligonucleotide therapy for a rare genetic disease. N Engl J Med. (2019) 381:1644–52. doi: 10.1056/NEJMoa1813279, PMID: 31597037 PMC6961983

[99] Gerk A Kundu S Meara JG Stegmann J. Digital solutions for rare diseases in global health. Lancet Glob Health. (2024) 12:e1091. doi: 10.1016/S2214-109X(24)00189-X, PMID: 38801829

[100] Senyel D Senn K Boyd J Nagels K. A systematic review of telemedicine for neuromuscular diseases: components and determinants of practice. BMC Digit Health. (2024) 2:17. doi: 10.1186/s44247-024-00078-9

[101] Pian JMY Owusu N Vitarello J Yan WX Lo AW Yu TW. How to pay for individualized genetic medicines. Nat Med. (2024) 30:1816–8. doi: 10.1038/s41591-024-03071-x, PMID: 38898122

[102] Fernández-Salido M Alhambra-Borrás T Casanova G Garcés-Ferrer J. Value-based healthcare delivery: a scoping review. Int J Environ Res Public Health. (2024) 21:134. doi: 10.3390/ijerph21020134, PMID: 38397625 PMC10888410

[103] Venema A Peeks F Rossi A Jager EA Derks TGJ. Towards values-based healthcare for inherited metabolic disorders: an overview of current practices for persons with liver glycogen storage disease and fatty acid oxidation disorders. J Inherit Metab Dis. (2022) 45:1018–27. doi: 10.1002/jimd.12555, PMID: 36088581 PMC9828459

[104] He W Li M Cao L Liu R You J Jing F. Introducing value-based healthcare perspectives into hospital performance assessment: a scoping review. J Evid-Based Med. (2023) 16:200–15. doi: 10.1111/jebm.12534, PMID: 37228246

[105] Fuchs B Heesen P SwissSarcomaNetwork. From data integration to precision medicine: a value-based healthcare approach for sarcoma care. J Clin Med. (2024). doi: 10.3390/jcm13216500, PMID: 39518639 PMC11546467

[106] Alnaqbi KA Elezbawy B Fasseeh AN Bangash AR Elshamy A Shendi H. Development of the emirates multi-criteria decision analysis tool for orphan drugs. Cureus. (2024) 16:e55215. doi: 10.7759/cureus.55215, PMID: 38558740 PMC10981202

[107] Teisberg E Wallace S O’Hara S. Defining and implementing value-based health care: a strategic framework. Acad Med. (2020) 95:682–5. doi: 10.1097/ACM.0000000000003122, PMID: 31833857 PMC7185050

[108] Bolislis WR Corriol-Rohou S Hill-Venning C Hoogland H Joos A King D. Orphan medicines for pediatric use: a focus on the European Union. Clin Ther. (2019) 41:2630–42. doi: 10.1016/j.clinthera.2019.10.006, PMID: 31704041

[109] Jones JT Morelli KA Vesely EM Puerner CTS Pavuluri CK Ross BS. The cystic fibrosis treatment Trikafta affects the growth, viability, and cell wall of *Aspergillus fumigatus* biofilms. MBio. (2023) 14. doi: 10.1128/mbio.01516-23, PMID: 37830825 PMC10653927

[110] Servais L Mercuri E Straub V Guglieri M Seferian AM Scoto M. Long-term safety and efficacy data of Golodirsen in ambulatory patients with Duchenne muscular dystrophy amenable to exon 53 skipping: a first-in-human, multicenter, two-part, open-label, phase 1/2 trial. Nucleic Acid Ther. (2022) 32:29–39. doi: 10.1089/nat.2021.0043, PMID: 34788571 PMC8817703

[111] Chambers JD Silver MC Berklein FC Cohen JT Neumann PJ. Orphan drugs offer larger health gains but less favorable cost-effectiveness than non-orphan drugs. J Gen Intern Med. (2020) 35:2629–36. doi: 10.1007/s11606-020-05805-2, PMID: 32291711 PMC7458970

[112] McGirr AA Schwartz KL Allen U Solomon M Sander B. The cost-effectiveness of palivizumab in infants with cystic fibrosis in the Canadian setting: a decision analysis model. Hum Vaccin Immunother. (2017) 13:599–606. doi: 10.1080/21645515.2016.1235670, PMID: 27768505 PMC5360124

[113] Toro W Yang M Georgieva M Song W Patel A Jiang AX. Health care resource utilization and costs for patients with spinal muscular atrophy: findings from a retrospective US claims database analysis. Adv Ther. (2023) 40:4589–605. doi: 10.1007/s12325-023-02621-y, PMID: 37587305 PMC10499678

[114] Mu Y Song K Song Y. A Cross-sectional study of Price and affordability of drugs for rare diseases in Shandong Province, China. Int J Environ Res Public Health. (2022) 19:13319. doi: 10.3390/ijerph192013319, PMID: 36293897 PMC9602851

[115] Villa F Di Filippo A Pierantozzi A Genazzani A Addis A Trifirò G. Orphan drug prices and epidemiology of rare diseases: a Cross-sectional study in Italy in the years 2014–2019. Front Med. (2022) 9:820757. doi: 10.3389/fmed.2022.820757, PMID: 35252257 PMC8891228

[116] Spagnolo P Du Bois RM. The challenges of clinical research in orphan diseases In: Cottin V Cordier JF Richeldi L, editors. Orphan lung diseases. London: Springer London (2015). 5–15.

[117] Whiting P Al M Burgers L Westwood M Ryder S Hoogendoorn M. Ivacaftor for the treatment of patients with cystic fibrosis and the G551D mutation: a systematic review and cost-effectiveness analysis. Health Technol Assess Winch Engl. (2014) 18:1–106. doi: 10.3310/hta18180, PMID: 24656117 PMC4780965

[118] Finkel RS Mercuri E Darras BT Connolly AM Kuntz NL Kirschner J. Nusinersen versus sham control in infantile-onset spinal muscular atrophy. N Engl J Med. (2017) 377:1723–32. doi: 10.1056/NEJMoa1702752, PMID: 29091570

[119] Ueda K Hokugo J. Safety and efficacy of idursulfase in the treatment of mucopolysaccharidosis II (Hunter syndrome): a post-marketing study in Japan. Expert Opin Drug Saf. (2020) 19:891–901. doi: 10.1080/14740338.2020.1751120, PMID: 32342708

[120] Zampoli M Verstraete J Frauendorf M Kassanjee R Workman L Morrow BM. Cystic fibrosis in South Africa: spectrum of disease and determinants of outcome. ERJ Open Res. (2021) 7:00856–2020. doi: 10.1183/23120541.00856-2020, PMID: 34350279 PMC8326682

[121] Ramalho ARO Ramalho RJR Oliveira CRP Magalhães MMGS Santos EG Sarmento PMP. Evaluation of effectiveness and outcome of PKU screening and management in the state of Sergipe. Brazil Arq Bras Endocrinol Metabol. (2014) 58:62–7. doi: 10.1590/0004-2730000002885, PMID: 24728166

[122] Angelis A Tordrup D Kanavos P. Socio-economic burden of rare diseases: a systematic review of cost of illness evidence. Health Policy. (2015) 119:964–79. doi: 10.1016/j.healthpol.2014.12.016, PMID: 25661982

[123] Chung CCY Ng NYT Ng YNC Lui ACY Fung JLF Chan MCY. Socio-economic costs of rare diseases and the risk of financial hardship: a cross-sectional study. Lancet Reg Health West Pac. (2023) 34:100711. doi: 10.1016/j.lanwpc.2023.100711, PMID: 37283971 PMC10240356

[124] Sequeira AR Mentzakis E Archangelidi O Paolucci F. The economic and health impact of rare diseases: a meta-analysis. Health Policy Technol. (2021) 10:32–44. doi: 10.1016/j.hlpt.2021.02.002

[125] Velvin G Dammann B Haagensen T Johansen H Strømme H Geirdal AØ. Work participation in adults with rare genetic diseases - a scoping review. BMC Public Health. 23:910. doi: 10.1186/s12889-023-15654-3, PMID: 37208707 PMC10197424

[126] Currie GR Gerber B Lorenzetti D MacDonald K Benseler SM Bernier FP. Developing a framework of cost elements of socioeconomic burden of rare disease: a scoping review. PharmacoEconomics. (2023) 41:803–18. doi: 10.1007/s40273-023-01262-x, PMID: 37029233

[127] Epps C Bax R Croker A Green D Gropman A Klein AV. Global regulatory and public health initiatives to advance pediatric drug development for rare diseases. Ther Innov Regul Sci. (2022) 56:964–75. doi: 10.1007/s43441-022-00409-w, PMID: 35471559 PMC9040360

[128] Durairaj C Bhattacharya I. Challenges, approaches and enablers: effectively triangulating towards dose selection in pediatric rare diseases. J Pharmacokinet Pharmacodyn. (2023) 50:445–59. doi: 10.1007/s10928-023-09868-6, PMID: 37296230

[129] Schreiber-Katz O Klug C Thiele S Schorling E Zowe J Reilich P. Comparative cost of illness analysis and assessment of health care burden of Duchenne and Becker muscular dystrophies in Germany. Orphanet J Rare Dis. (2014) 9:210. doi: 10.1186/s13023-014-0210-9, PMID: 25519771 PMC4302713

[130] Angelis A Kanavos P López-Bastida J Linertová R Oliva-Moreno J Serrano-Aguilar P. Social/economic costs and health-related quality of life in patients with epidermolysis bullosa in Europe. Eur J Health Econ HEPAC Health Econ Prev Care. (2016) 17:31–42. doi: 10.1007/s10198-016-0783-4, PMID: 27107597 PMC4869727

[131] López-Bastida J Oliva-Moreno J Linertová R Serrano-Aguilar P. Social/economic costs and health-related quality of life in patients with rare diseases in Europe. Eur J Health Econ. (2016) 17:1–5. doi: 10.1007/s10198-016-0780-7, PMID: 27023708

[132] Iskrov G Astigarraga I Stefanov R López-Bastida J Linertová R Oliva-Moreno J. Social/economic costs and health-related quality of life in patients with histiocytosis in Europe. Eur J Health Econ HEPAC Health Econ Prev Care. (2016) 17:67–78. doi: 10.1007/s10198-016-0790-5, PMID: 27042831

[133] Yang G Cintina I Pariser A Oehrlein E Sullivan J Kennedy A. The national economic burden of rare disease in the United States in 2019. Orphanet J Rare Dis. (2022) 17:163. doi: 10.1186/s13023-022-02299-5, PMID: 35414039 PMC9004040

[134] Dias AG Daher A Barrera Ortiz L Carreño-Moreno S Hafez HSR Jansen AM. Rarecare: a policy perspective on the burden of rare diseases on caregivers in Latin America. Front Public Health. (2023) 11:1127713. doi: 10.3389/fpubh.2023.1127713, PMID: 36935700 PMC10017724

[135] Luzzatto L Makani J. Treating rare diseases in Africa: the drugs exist but the need is unmet. Front Pharmacol. (2022) 12:770640. doi: 10.3389/fphar.2021.770640, PMID: 35082665 PMC8784510

[136] Heinrich F Cordts I Günther R Stolte B Zeller D Schröter C. Economic evaluation of motor neuron diseases: a nationwide cross-sectional analysis in Germany. J Neurol. (2023) 270:4922–38. doi: 10.1007/s00415-023-11811-1, PMID: 37356024 PMC10511618

[137] Rombach SM Hollak CE Linthorst GE Dijkgraaf MG. Cost-effectiveness of enzyme replacement therapy for Fabry disease. Orphanet J Rare Dis. (2013) 8:29. doi: 10.1186/1750-1172-8-29, PMID: 23421808 PMC3598841

[138] McMillan HJ Gerber B Cowling T Khuu W Mayer M Wu JW. Burden of spinal muscular atrophy (SMA) on patients and caregivers in Canada. J Neuromuscul Dis. (2021) 8:553–68. doi: 10.3233/JND-200610, PMID: 33749617 PMC8385498

[139] Kodra Y Cavazza M Schieppati A De Santis M Armeni P Arcieri R. The social burden and quality of life of patients with haemophilia in Italy. Blood Transfus. (2014) 12:s567–75. doi: 10.2450/2014.0042-14s, PMID: 24922297 PMC4044804

[140] Rodriguez-Santana I DasMahapatra P Burke T Hakimi Z Bartelt-Hofer J Nazir J. Health-related quality of life, direct medical and societal costs among children with moderate or severe haemophilia in Europe: multivariable models of the CHESS-PAEDs study. Orphanet J Rare Dis. (2022) 17:150. doi: 10.1186/s13023-022-02301-0, PMID: 35379284 PMC8981697

[141] López-Bastida J Linertová R Oliva-Moreno J Serrano-Aguilar P Posada-de-la-Paz M Kanavos P. Social/economic costs and health-related quality of life in patients with scleroderma in Europe. Eur J Health Econ HEPAC Health Econ Prev Care. (2016) 17:109–17. doi: 10.1007/s10198-016-0789-y, PMID: 27038626

[142] López-Bastida J Linertová R Oliva-Moreno J Posada-de-la-Paz M Serrano-Aguilar P. Social economic costs and health-related quality of life in patients with systemic sclerosis in Spain. Arthritis Care Res. (2014) 66:473–80. doi: 10.1002/acr.22167, PMID: 24022829

[143] Leso V Romano R Santocono C Caruso M Iacotucci P Carnovale V. The impact of cystic fibrosis on the working life of patients: a systematic review. J Cyst Fibros. (2022) 21:361–9. doi: 10.1016/j.jcf.2021.08.011, PMID: 34470710

[144] Frey S Stargardt T Schneider U Schreyögg J. The economic burden of cystic fibrosis in Germany from a payer perspective. PharmacoEconomics. (2019) 37:1029–39. doi: 10.1007/s40273-019-00797-2 30949989

[145] Thorat T McGarry LJ Bonafede MM Limone BL Rubin JL Jariwala-Parikh K. Healthcare resource utilization and costs among children with cystic fibrosis in the United States. Pediatr Pulmonol. (2021) 56:2833–44. doi: 10.1002/ppul.25535, PMID: 34138523 PMC8456795

[146] Chambers GM Settumba SN Carey KA Cairns A Menezes MP Ryan M. Prenusinersen economic and health-related quality of life burden of spinal muscular atrophy. Neurology. (2020) 95:e1–e10. doi: 10.1212/WNL.0000000000009715, PMID: 32513788

[147] López-Bastida J Peña-Longobardo LM Aranda-Reneo I Tizzano E Sefton M Oliva-Moreno J. Social/economic costs and health-related quality of life in patients with spinal muscular atrophy (SMA) in Spain. Orphanet J Rare Dis. (2017) 12:141. doi: 10.1186/s13023-017-0695-0, PMID: 28821278 PMC5563035

[148] Klug C Schreiber-Katz O Thiele S Schorling E Zowe J Reilich P. Disease burden of spinal muscular atrophy in Germany. Orphanet J Rare Dis. (2016) 11:58. doi: 10.1186/s13023-016-0424-0, PMID: 27145956 PMC4857429

[149] Teoh LJ Geelhoed EA Bayley K Leonard H Laing NG. Health care utilization and costs for children and adults with duchenne muscular dystrophy. Muscle Nerve. (2016) 53:877–84. doi: 10.1002/mus.24965, PMID: 26562484

[150] Qi X Xu J Shan L Li Y Cui Y Liu H. Economic burden and health related quality of life of ultra-rare Gaucher disease in China. Orphanet J Rare Dis. (2021) 16:358. doi: 10.1186/s13023-021-01963-6, PMID: 34380529 PMC8356434

[151] Mhatre SP Muranjan M Gogtay NJ. Economic burden of Gaucher disease at a tertiary care public Hospital in Mumbai. Indian J Pediatr. (2024) 91:463–9. doi: 10.1007/s12098-023-04740-4, PMID: 37486590

[152] Uchil A Muranjan M Gogtay NJ. Economic burden of beta-thalassaemia major receiving hypertransfusion therapy at a public hospital in Mumbai. Natl Med J India. (2023) 36:11–6. doi: 10.25259/NMJI_580_20, PMID: 37615146

[153] Shafie AA Wong JHY Ibrahim HM Mohammed NS Chhabra IK. Economic burden in the management of transfusion-dependent thalassaemia patients in Malaysia from a societal perspective. Orphanet J Rare Dis. (2021) 16:157. doi: 10.1186/s13023-021-01791-8, PMID: 33827621 PMC8028190

[154] Chevreul K Berg Brigham K Clément MC Poitou C Tauber M. Members of the BURQOL-RD research network listed in the online appendix. Economic burden and health-related quality of life associated with Prader-Willi syndrome in France. J Intellect Disabil Res JIDR. (2016) 60:879–90. doi: 10.1111/jir.12288, PMID: 27174598

[155] Shoffstall AJ Gaebler JA Kreher NC Niecko T Douglas D Strong TV. The high direct medical costs of Prader-Willi syndrome. J Pediatr. (2016) 175:137–43. doi: 10.1016/j.jpeds.2016.05.018, PMID: 27283463 PMC7464637

[156] Stella P Gold-von SG. Pharmaceutical pricing, cost containment and new treatments for rare diseases in children. Orphanet J Rare Dis. (2014) 9:152. doi: 10.1186/s13023-014-0152-2, PMID: 25348640 PMC4221662

[157] Vadagam P Kamal KM. Hospitalization costs of cystic fibrosis in the United States: a retrospective analysis. Hosp Pract. (1995) 46:203–13. doi: 10.1080/21548331.2018.1505407, PMID: 30067115

[158] Kodra Y Cavazza M de Santis M Guala A Liverani ME Armeni P. Social economic costs, health-related quality of life and disability in patients with cri Du chat syndrome. Int J Environ Res Public Health. (2020) 17:5951. doi: 10.3390/ijerph17165951, PMID: 32824402 PMC7459640

[159] Rose AM Grosse SD Garcia SP Bach J Kleyn M Simon NJE. The financial and time burden associated with phenylketonuria treatment in the United States. Mol Genet Metab Rep. (2019) 21:100523. doi: 10.1016/j.ymgmr.2019.100523, PMID: 31660292 PMC6807265

[160] Rajmil L Perestelo-Pérez L Herdman M. Quality of life and rare diseases In: Posada De La Paz M Groft SC, editors. Rare diseases epidemiology, vol. 686. Dordrecht: Springer Netherlands (2010). 251–72. 10.1007/978-90-481-9485-8_15 20824450

[161] Hurley N Moyna NM Kehoe B McCaffrey N Redmond K Hardcastle SJ. Factors influencing physical activity in adults with cystic fibrosis. BMC Pulm Med. (2021) 21:113. doi: 10.1186/s12890-021-01482-x, PMID: 33810783 PMC8017094

[162] Quinn L Playle R Drew CJG Taiyari K Williams-Thomas R Muratori LM. Physical activity and exercise outcomes in Huntington’s disease (PACE-HD): results of a 12-month trial-within-cohort feasibility study of a physical activity intervention in people with Huntington’s disease. Parkinsonism Relat Disord. (2022) 101:75–89. doi: 10.1016/j.parkreldis.2022.06.013, PMID: 35809488

[163] Vitale SA. Individual and family issues surrounding a rare disease. Clin Nurs Stud. (2017) 6:45. doi: 10.5430/cns.v6n1p45

[164] Kenny T Bogart K Freedman A Garthwaite C Henley S Bolz-Johnson M. The importance of psychological support for parents and caregivers of children with a rare disease at diagnosis. Rare Dis Orphan Drugs J. (2022) 1:7. doi: 10.20517/rdodj.2022.04

[165] Schuller Y Hollak CEM Biegstraaten M. The quality of economic evaluations of ultra-orphan drugs in Europe – a systematic review. Orphanet J Rare Dis. (2015) 10:92. doi: 10.1186/s13023-015-0305-y, PMID: 26223689 PMC4520069

[166] Cannizzo S Lorenzoni V Palla I Pirri S Trieste L Triulzi I. Rare diseases under different levels of economic analysis: current activities, challenges and perspectives. RMD Open. (2018) 4:e000794. doi: 10.1136/rmdopen-2018-000794, PMID: 30488003 PMC6241967

[167] Zurynski Y Gonzalez A Deverell M Phu A Leonard H Christodoulou J. Rare disease: a national survey of paediatricians’ experiences and needs. BMJ Paediatr Open. (2017) 1:e000172. doi: 10.1136/bmjpo-2017-000172, PMID: 29637168 PMC5862166

[168] Gómez-Zúñiga B Pulido R Pousada M Armayones M. The role of parent/caregiver with children affected by rare diseases: navigating between love and fear. Int J Environ Res Public Health. (2021) 18:3724. doi: 10.3390/ijerph18073724, PMID: 33918362 PMC8038217

[169] Currie G Szabo J. Social isolation and exclusion: the parents’ experience of caring for children with rare neurodevelopmental disorders. Int J Qual Stud Health Well-Being. (2020) 15:1725362. doi: 10.1080/17482631.2020.1725362, PMID: 32048917 PMC7034477

[170] Bogart K Hemmesch A Barnes E Blissenbach T Beisang A Engel P. Healthcare access, satisfaction, and health-related quality of life among children and adults with rare diseases. Orphanet J Rare Dis. (2022) 17:196. doi: 10.1186/s13023-022-02343-4, PMID: 35549731 PMC9096775

[171] Atkins JC Padgett CR. Living with a rare disease: psychosocial impacts for parents and family members – a systematic review. J Child Fam Stud. (2024) 33:617–36. doi: 10.1007/s10826-024-02790-6

[172] Robards F Kang M Luscombe G Hawke C Sanci L Steinbeck K. Intersectionality: social marginalisation and self-reported health status in young people. Int J Environ Res Public Health. (2020) 17:8104. doi: 10.3390/ijerph17218104, PMID: 33153094 PMC7663617

[173] Paz-Lourido B Negre F De La Iglesia B Verger S. Influence of schooling on the health-related quality of life of children with rare diseases. Health Qual Life Outcomes. (2020) 18:109. doi: 10.1186/s12955-020-01351-x, PMID: 32345307 PMC7189684

[174] Adama EA Arabiat D Foster MJ Afrifa-Yamoah E Runions K Vithiatharan R. The psychosocial impact of rare diseases among children and adolescents attending mainstream schools in Western Australia. Int J Incl Educ. (2023) 27:1273–86. doi: 10.1080/13603116.2021.1888323

[175] Foster M Adama E Arabiat D Runions K Vithiatharan R Zgambo M. Parents’ experiences of children with a rare disease attending a mainstream school: Australia. J Pediatr Nurs. (2022) 63:e50–7. doi: 10.1016/j.pedn.2021.10.013, PMID: 34716060

[176] Baynam G Gomez R Jain R. Stigma associated with genetic testing for rare diseases—causes and recommendations. Front Genet. (2024) 15:1335768. doi: 10.3389/fgene.2024.1335768, PMID: 38638122 PMC11024281

[177] Puiu M Dan D. Rare diseases, from European resolutions and recommendations to actual measures and strategies. Maedica. (2010) 5:128–31. PMID: 21977136 PMC3150005

[178] Evans WR Rafi I. Rare diseases in general practice: recognising the zebras among the horses. Br J Gen Pract. (2016) 66:550–1. doi: 10.3399/bjgp16X687625, PMID: 27789486 PMC5072891

[179] Lenderking WR Anatchkova M Pokrzywinski R Skalicky A Martin ML Gelhorn H. Measuring health-related quality of life in patients with rare disease. J Patient Rep Outcomes. (2021) 5:61. doi: 10.1186/s41687-021-00336-8, PMID: 34283357 PMC8292508

[180] Vandeleur M Walter LM Armstrong DS Robinson P Nixon GM Horne RSC. Quality of life and mood in children with cystic fibrosis: associations with sleep quality. J Cyst Fibros Off J Eur Cyst Fibros Soc. (2018) 17:811–20. doi: 10.1016/j.jcf.2017.11.021, PMID: 29277313

[181] Pelentsov LJ Fielder AL Laws TA Esterman AJ. The supportive care needs of parents with a child with a rare disease: results of an online survey. BMC Fam Pract. (2016) 17:88. doi: 10.1186/s12875-016-0488-x, PMID: 27439905 PMC4955113

[182] Mcmullan J Lohfeld L McKnight AJ. Needs of informal caregivers of people with a rare disease: a rapid review of the literature. BMJ Open. (2022) 12:e063263. doi: 10.1136/bmjopen-2022-063263, PMID: 36523233 PMC9748923

[183] Swoboda KJ Nery FC. Harnessing the power of the patient perspective for rare disease therapeutics. Neurology. (2018) 91:585–6. doi: 10.1212/WNL.0000000000006230, PMID: 30143562

[184] Togo CCG Zidorio APC Gonçalves VSS Hubbard L de Carvalho KMB Dutra ES. Quality of life in people with epidermolysis bullosa: a systematic review. Qual Life Res Int J Qual Life Asp Treat Care Rehabil. (2020) 29:1731–45. doi: 10.1007/s11136-020-02495-5, PMID: 32246433

[185] Reimer A Bruckner-Tuderman L Ott H. Mapping health care of rare diseases: the example of epidermolysis bullosa in Germany. Orphanet J Rare Dis. (2018) 13:197. doi: 10.1186/s13023-018-0944-x, PMID: 30409176 PMC6225719

[186] Ryder S Leadley RM Armstrong N Westwood M de Kock S Butt T. The burden, epidemiology, costs and treatment for Duchenne muscular dystrophy: an evidence review. Orphanet J Rare Dis. (2017) 12:79. doi: 10.1186/s13023-017-0631-3, PMID: 28446219 PMC5405509

[187] Rozensztrauch A Śmigiel R. Quality of life in children with Prader–Willi syndrome and the impact of the disease on the functioning of families. Int J Environ Res Public Health. (2022) 19:16330. doi: 10.3390/ijerph192316330, PMID: 36498413 PMC9740001

[188] Belzer LT Wright SM Goodwin EJ Singh MN Carter BS. Psychosocial considerations for the child with rare disease: a review with recommendations and calls to action. Children. (2022) 9:933. doi: 10.3390/children9070933, PMID: 35883917 PMC9325007

[189] Adams D Clarke S Griffith G Howlin P Moss J Petty J. Mental health and well-being in mothers of children with rare genetic syndromes showing chronic challenging behavior: a Cross-sectional and longitudinal study. Am J Intellect Dev Disabil. (2018) 123:241–53. doi: 10.1352/1944-7558-123.3.241, PMID: 29671635

[190] Quittner AL Goldbeck L Abbott J Duff A Lambrecht P Solé A. Prevalence of depression and anxiety in patients with cystic fibrosis and parent caregivers: results of the international depression epidemiological study across nine countries. Thorax. (2014) 69:1090–7. doi: 10.1136/thoraxjnl-2014-205983, PMID: 25246663

[191] Maltseva M Schubert-Bast S Zöllner JP Bast T Mayer T Von Spiczak S. Sleep quality, anxiety, symptoms of depression, and caregiver burden among those caring for patients with Dravet syndrome: a prospective multicenter study in Germany. Orphanet J Rare Dis. (2023) 18:98. doi: 10.1186/s13023-023-02697-3, PMID: 37120555 PMC10148440

[192] Domaradzki J Walkowiak D. Caring for children with Dravet syndrome: exploring the daily challenges of family caregivers. Children. (2023) 10:1410. doi: 10.3390/children10081410, PMID: 37628409 PMC10453293

[193] Boettcher J Denecke J Barkmann C Wiegand-Grefe S. Quality of life and mental health in mothers and fathers caring for children and adolescents with rare diseases requiring long-term mechanical ventilation. Int J Environ Res Public Health. (2020) 17:8975. doi: 10.3390/ijerph17238975, PMID: 33276595 PMC7731445

[194] Rezaei F Sanagoo A Peyrovi H Jouybari L. Persistent suffering: living experiences of patients with rare disease: an interpretative phenomenological study. J Educ Health Promot. (2023) 12:224. doi: 10.4103/jehp.jehp_1010_22, PMID: 37727410 PMC10506786

[195] Danese E Lippi G. Rare diseases: the paradox of an emerging challenge. Ann Transl Med. (2018) 6:329. doi: 10.21037/atm.2018.09.04, PMID: 30306068 PMC6174191

[196] Verger S Negre F Fernández-Hawrylak M Paz-Lourido B. The impact of the coordination between healthcare and educational personnel on the health and inclusion of children and adolescents with rare diseases. Int J Environ Res Public Health. (2021) 18:6538. doi: 10.3390/ijerph18126538, PMID: 34204503 PMC8296368

[197] García-Perales R Palomares-Ruiz A Ordóñez-García L García-Toledano E. Rare diseases in the educational field: knowledge and perceptions of Spanish teachers. Int J Environ Res Public Health. (2022) 19:6057. doi: 10.3390/ijerph19106057, PMID: 35627593 PMC9140519

[198] Branch-Smith C Shaw T Lin A Runions K Payne D Nguyen R. Bullying and mental health amongst Australian children and young people with cystic fibrosis. Am J Orthopsychiatry. (2018) 88:402–12. doi: 10.1037/ort0000289, PMID: 29999388

[199] Röjvik A Jaeger G Hjelmquist E Falkman KW. Creating optimal educational settings for children with rare diseases – a working method. Eur J Spec Needs Educ. (2024) 39:759–73. doi: 10.1080/08856257.2023.2294237

[200] Omosigho PO John OO Musa MB Aboelhassan YMEI Olabode ON Bouaddi O. Stigma and infectious diseases in Africa: examining impact and strategies for reduction. Ann Med Surg. (2023) 85:6078–82. doi: 10.1097/MS9.0000000000001470, PMID: 38098545 PMC10718398

[201] Depping MK Uhlenbusch N von Kodolitsch Y Klose HFE Mautner VF Löwe B. Supportive care needs of patients with rare chronic diseases: multi-method, cross-sectional study. Orphanet J Rare Dis. (2021) 16:44. doi: 10.1186/s13023-020-01660-w, PMID: 33482869 PMC7825171

[202] Gómez-Díaz B Zamora-González EO Miranda-Duarte A Roque-Ramírez B Vázquez-Cárdenas NA Martínez-Gómez G. Assessing knowledge, perceptions, awareness and attitudes on rare diseases among health care providers and health students in Mexico. Rare. (2023) 1:100005. doi: 10.1016/j.rare.2023.100005, PMID: 40052061

[203] Lyon ME Wiener L. Special issue: psychosocial considerations for children and adolescents living with a rare disease. Children. (2022) 9:1099. doi: 10.3390/children9071099, PMID: 35884083 PMC9322344

[204] Domaradzki J Walkowiak D. Ultra-rare ultra-care: assessing the impact of caring for children with ultra rare diseases. Eur J Paediatr Neurol EJPN Off J Eur Paediatr Neurol Soc. (2024) 48:78–84. doi: 10.1016/j.ejpn.2023.12.003, PMID: 38071849

[205] Spurr S Danford CA Roberts KJ Sheppard-LeMoine D Machado Silva-Rodrigues F Darezzo Rodrigues Nunes M. Fathers’ experiences of caring for a child with a chronic illness: a systematic review. Child Basel Switz. (2023) 10:197. doi: 10.3390/children10020197, PMID: 36832326 PMC9955404

[206] Daly C Ruane P O’Reilly K Longworth L Vega-Hernandez G. Caregiver burden in cystic fibrosis: a systematic literature review. Ther Adv Respir Dis. (2022) 16:17534666221086416. doi: 10.1177/17534666221086416, PMID: 35323061 PMC8958690

[207] Soelaeman RH Smith MG Sahay K Tilford JM Goodenough D Paramsothy P. Labor market participation and productivity costs for female caregivers of minor male children with Duchenne and Becker muscular dystrophies. Muscle Nerve. (2021) 64:717–25. doi: 10.1002/mus.27429, PMID: 34605048 PMC8595851

[208] Khosla N Valdez R. A compilation of national plans, policies and government actions for rare diseases in 23 countries. Intractable Rare Dis Res. (2018) 7:213–22. doi: 10.5582/irdr.2018.01085, PMID: 30560012 PMC6290840

[209] Lopes-Júnior LC Ferraz VEF Lima RAG Schuab SIPC Pessanha RM Luz GS. Health policies for rare disease patients: a scoping review. Int J Environ Res Public Health. (2022) 19:15174. doi: 10.3390/ijerph192215174, PMID: 36429893 PMC9690117

[210] United Nations General Assembly. (2021). Available online at: https://documents.un.org/doc/undoc/gen/n23/420/62/pdf/n2342062.pdf (Accessed January 15, 2025).

[211] O’Mahony B Dolan G Nugent D Goodman C. The international Haemophilia access strategy council. Patient Centred Value Framework Haemophilia Haemophilia. (2018) 24:873–9. doi: 10.1111/hae.13456 29626368

[212] Choudhury MC Saberwal G. The role of patient organizations in the rare disease ecosystem in India: an interview based study. Orphanet J Rare Dis. (2019) 14:117. doi: 10.1186/s13023-019-1093-6, PMID: 31142331 PMC6542017

[213] CCY C Hong Kong Genome Project ATW C BHY C. Rare disease emerging as a global public health priority. Front. Public Health. (2022) 10:1028545. doi: 10.3389/fpubh.2022.1028545, PMID: 36339196 PMC9632971

[214] Mikami K. Orphans in the market: the history of orphan drug policy. Soc Hist Med. (2019) 32:609–30. doi: 10.1093/shm/hkx098, PMID: 31384102 PMC6664588

[215] Montserrat A Taruscio D. Policies and actions to tackle rare diseases at European level. Ann Ist Super Sanita. (2019) 55:296–304. doi: 10.4415/ANN_19_03_17, PMID: 31553326

[216] Wainstock D Katz A. Advancing rare disease policy in Latin America: a call to action. Lancet Reg Health Am. (2023) 18:100434. doi: 10.1016/j.lana.2023.100434, PMID: 36844013 PMC9950655

[217] European Reference Networks - European Commission. (2024). Available online at: https://health.ec.europa.eu/rare-diseases-and-european-reference-networks/european-reference-networks_en (Accessed January 15, 2025).

[218] Boulanger V Schlemmer M Rossov S Seebald A Gavin P. Establishing patient registries for rare diseases: rationale and challenges. Pharm Med. (2020) 34:185–90. doi: 10.1007/s40290-020-00332-1, PMID: 32215853 PMC7286934

[219] Mishra S Bhat D Venkatesh MP. Navigating health policies and programs in India: exploring opportunities to improve rare disease management and orphan drug research. Orphanet J Rare Dis. (2024) 19:446. doi: 10.1186/s13023-024-03377-6, PMID: 39614301 PMC11606027

[220] Grand TS Ren S Hall J Åström DO Regnier S Thokala P. Issues, challenges and opportunities for economic evaluations of orphan drugs in rare diseases: an umbrella review. PharmacoEconomics. (2024) 42:619–31. doi: 10.1007/s40273-024-01370-2, PMID: 38616217 PMC11126517

[221] Witt S Schuett K Wiegand-Grefe S Boettcher J Quitmann J. Living with a rare disease - experiences and needs in pediatric patients and their parents. Orphanet J Rare Dis. (2023) 18:242. doi: 10.1186/s13023-023-02837-9, PMID: 37568186 PMC10422846

[222] Auvin S Irwin J Abi-Aad P Battersby A. The problem of rarity: estimation of prevalence in rare disease. Value Health. (2018) 21:501–7. doi: 10.1016/j.jval.2018.03.002, PMID: 29753345

[223] Julkowska D Austin CP Cutillo CM Gancberg D Hager C Halftermeyer J. The importance of international collaboration for rare diseases research: a European perspective. Gene Ther. (2017) 24:562–71. doi: 10.1038/gt.2017.29, PMID: 28440796 PMC5628265

[224] García-Pérez L Linertová R Valcárcel-Nazco C Posada M Gorostiza I Serrano-Aguilar P. Cost-of-illness studies in rare diseases: a scoping review. Orphanet J Rare Dis. (2021) 16:178. doi: 10.1186/s13023-021-01815-3, PMID: 33849613 PMC8045199

[225] Ngoumou RD Feudjio YBD. The management of rare disease patients from a grassroot perspective: the role of patients’ organizations in the global recognition of rare diseases in Cameroon. Pan Afr Med J. (2024) 47:64. doi: 10.11604/pamj.2024.47.64.38226, PMID: 38681114 PMC11055193

[226] Edu E Okesanya OJ Lucero-Prisno DE. Burden of rare diseases in Africa: recommendations for improving access to medications and healthcare. J Med Surg Public Health. (2024) 2:100032. doi: 10.1016/j.glmedi.2023.100032, PMID: 40052061

[227] Baynam G Julkowska D Bowdin S Hermes A McMaster CR Prichep E. Advancing diagnosis and research for rare genetic diseases in indigenous peoples. Nat Genet. (2024) 56:189–93. doi: 10.1038/s41588-023-01642-1, PMID: 38332370 PMC11229440

[228] Mudau MM Seymour H Nevondwe P Kerr R Spencer C Feben C. A feasible molecular diagnostic strategy for rare genetic disorders within resource-constrained environments. J Community Genet. (2024) 15:39–48. doi: 10.1007/s12687-023-00674-8, PMID: 37815686 PMC10858011

[229] Schneider NB Roos EC Staub ALP Bevilacqua IP de Almeida AC de Camargo MT. Estimated costs for Duchenne muscular dystrophy care in Brazil. Orphanet J Rare Dis. (2023) 18:159. doi: 10.1186/s13023-023-02767-6, PMID: 37349725 PMC10288739

[230] Lumaka A Carstens N Devriendt K Krause A Kulohoma B Kumuthini J. Increasing African genomic data generation and sharing to resolve rare and undiagnosed diseases in Africa: a call-to-action by the H3Africa rare diseases working group. Orphanet J Rare Dis. (2022) 17:230. doi: 10.1186/s13023-022-02391-w, PMID: 35710439 PMC9201791

[231] Shah KK Kogut S Slitt A. Challenges in evaluating safety and efficacy in drug development for rare diseases: a review for pharmacists. J Pharm Pract. (2021) 34:472–9. doi: 10.1177/0897190020930972, PMID: 32583733

[232] Economist Impact. Perspectives. One stripe at a time: raising awareness of rare diseases in Latin America. Available online at: https://impact.economist.com/perspectives/health/one-stripe-time-raising-awareness-rare-diseases-latin-america (Accessed January 15, 2025).

[233] Simpson A Bloom L Fulop NJ Hudson E Leeson-Beevers K Morris S. How are patients with rare diseases and their carers in the UK impacted by the way care is coordinated? An exploratory qualitative interview study. Orphanet J Rare Dis. (2021) 16:76. doi: 10.1186/s13023-020-01664-6, PMID: 33568181 PMC7874609

[234] Li X Liu M Lin J Li B Zhang X Zhang S. A questionnaire-based study to comprehensively assess the status quo of rare disease patients and care-givers in China. Orphanet J Rare Dis. (2021) 16:327. doi: 10.1186/s13023-021-01954-7, PMID: 34294091 PMC8296703

[235] Domaradzki J Walkowiak D. Evaluating the challenges and needs of parents caring for children with Williams syndrome: a preliminary study from Poland. Res Dev Disabil. (2024) 145:104669. doi: 10.1016/j.ridd.2024.104669, PMID: 38215502

[236] Teutsch S Zurynski Y Eslick GD Deverell M Christodoulou J Leonard H. Australian children living with rare diseases: health service use and barriers to accessing care. World J Pediatr WJP. (2023) 19:701–9. doi: 10.1007/s12519-022-00675-6, PMID: 36653598 PMC9848027

[237] Velvin G Johnsen V Lidal IB Berg E. Parental intervention program for preschool children with rare diseases - a mixed methods evaluation of parents’ experiences and utility. Orphanet J Rare Dis. (2023) 18:327. doi: 10.1186/s13023-023-02935-8, PMID: 37848938 PMC10583464

[238] Meregaglia M Nicod E Drummond M. The estimation of health state utility values in rare diseases: overview of existing techniques. Int J Technol Assess Health Care. (2020) 36:469–73. doi: 10.1017/S0266462320000665, PMID: 32981547

[239] Korth-Bradley JM. Regulatory framework for drug development in rare diseases. J Clin Pharmacol. (2022) 62:S15–26. doi: 10.1002/jcph.2171, PMID: 36461739

[240] SYa V Nikolaeva EA Sokolov AA Kopishinskaia SV Gamirova RG Khalmatova BT. Children with rare diseases: ethical, social, psychological and medical issues. Ross Vestn Perinatol Pediatr Russ Bull Perinatol Pediatr. (2019) 64:149–54. doi: 10.21508/1027-4065-2019-64-5-149-154

[241] Abdallah K Claes K Huys I Follon L Calis C Simoens S. Exploring alternative financing models and early access schemes for orphan drugs: a Belgian case study. Orphanet J Rare Dis. (2022) 17:429. doi: 10.1186/s13023-022-02571-8, PMID: 36494733 PMC9733299

[242] About the DDRMD. (2025). Available online at: https://www.ddrmd.nl/ (Accessed January 15, 2025).

[243] Jonker CJ de Vries ST van den Berg HM McGettigan P Hoes AW Mol PGM. Capturing data in rare disease registries to support regulatory decision making: a survey study among industry and other stakeholders. Drug Saf. (2021) 44:853–61. doi: 10.1007/s40264-021-01081-z, PMID: 34091881 PMC8279983

[244] Simoens S. Pricing and reimbursement of orphan drugs: the need for more transparency. Orphanet J Rare Dis. (2011) 6:42. doi: 10.1186/1750-1172-6-42, PMID: 21682893 PMC3132155

[245] Scherman D Fétro C. Drug repositioning for rare diseases: knowledge-based success stories. Therapie. (2020) 75:161–7. doi: 10.1016/j.therap.2020.02.007, PMID: 32164975

[246] Alonso Ruiz A Large K Moon S Vieira M. Pharmaceutical policy and innovation for rare diseases: a narrative review. F1000Research. (2023) 12:211. doi: 10.12688/f1000research.130809.2, PMID: 38778810 PMC11109548

[247] Seifert O. HealthLumen. (2024). Available online at: https://www.healthlumen.com/supporting-rare-disease-patient-advocacy-with-epidemiological-insights/ (Accessed January 15, 2025).

[248] Global Partnerships. National organization for rare disorders. (2022). Available online at: https://rarediseases.org/community-support/global-partnerships/ (Accessed January 14, 2025).

[249] Héon-Klin V. European reference networks for rare diseases: what is the conceptual framework? Orphanet J Rare Dis. (2017) 12:137. doi: 10.1186/s13023-017-0676-3, PMID: 28784158 PMC5547471

[250] Hedley V Bolz-Johnson M Hernando I Kenward R Nabbout R Romero C. Together4RD position statement on collaboration between European reference networks and industry. Orphanet J Rare Dis. (2023) 18:272. doi: 10.1186/s13023-023-02853-9, PMID: 37670358 PMC10478454

[251] dos Santos VB Bernabé CH Zhang S Abaza H Benis N Cámara A. Towards FAIRification of sensitive and fragmented rare disease patient data: challenges and solutions in European reference network registries. Orphanet J Rare Dis. (2022) 17:436. doi: 10.1186/s13023-022-02558-5, PMID: 36517834 PMC9749345

